# N7-methylguanosin regulators-mediated methylation modification patterns and characterization of the immune microenvironment in lower-grade glioma

**DOI:** 10.1186/s40001-023-01108-4

**Published:** 2023-03-30

**Authors:** Aierpati Maimaiti, Zhaohai Feng, Yanwen Liu, Mirzat Turhon, Zhihao Xie, Yilimire Baihetiyaer, Xixian Wang, Maimaitijiang Kasimu, Lei Jiang, Yongxin Wang, Zengliang Wang, Yinan Pei

**Affiliations:** 1 Department of Neurosurgery, Neurosurgery Centre, The First Affiliated Hospital of Xinjiang Medical University, No. 137, South Liyushan Road, Xinshi District, Urumqi, 830054 Xinjiang China; 2 Department of Medical Laboratory, Xinjiang Production and Construction Corps Hospital, Urumqi, 830002 Xinjiang China; 3 Department of Neurointerventional Surgery, Beijing Neurosurgical Institute, Capital Medical University, Beijing, 100070 China; 4 Department of Neurointerventional Surgery, Beijing Tiantan Hospital, Capital Medical University, Beijing, 100070 China; 5 The Second Hospital of Jilin University, Changchun, 130041 Jilin China; 6 Department of Neurology, The First Affiliated Hospital of Xinjiang Medical University, Urumqi, 830054 Xinjiang China; 7 People’s Hospital of Mongolian Autonomous Prefecture of Bayingolin, Korla, 841000 Xinjiang China

**Keywords:** N7-methylguanosine, Anti-PD-1/L1 immunotherapy, Lower-grade glioma, Prognostic signature, Tumor microenvironment

## Abstract

N7-methylguanosine (m7G) modification signature has recently emerged as a crucial regulator of tumor progression and treatment in cancer. However, there is limited information available on the genomic profile of lower-grade gliomas (LGGs) related to m7G methylation modification genes’ function in tumorigenesis and progression. In this study, we employed bioinformatics methods to characterize m7G modifications in individuals with LGG from The Chinese Glioma Genome Atlas (CGGA) and The Cancer Genome Atlas (TCGA). We used gene set enrichment analysis (GSEA), single sample GSEA (ssGSEA), CIBERSORT algorithm, ESTIMATE algorithm, and TIDE to evaluate the association between m7G modification patterns, tumor microenvironment (TME) cell infiltration properties, and immune infiltration markers. The m7G scoring scheme using principal component analysis (PCA) was employed to investigate the m7G modification patterns quantitatively. We examined the m7G modification hub genes' expression levels in normal samples, refractory epilepsy samples, and LGG samples using immunohistochemistry, western-blotting, and qRT-PCR. Our findings revealed that individuals with LGG could be categorized into two groups based on m7G scores (high and low) according to the properties of m7G. Moreover, we observed that high m7G score was associated with significant clinical benefit and prolonged survival duration in the anti-PD-1 cohort, while low m7G score was associated with improved prognostic outcomes and increased likelihood of complete or partial response in the anti-PD-L1 cohort. Different m7G subtypes also showed varying Tumor Mutational Burden (TMB) and immune profiles and might have distinct responses to immunotherapy. Furthermore, we identified five potential genetic markers that were highly correlated with the m7G score signature index. These findings provide insight into the features and classification associated with m7G methylation modifications and may aid in improving the clinical outcome of LGG.

**Supplementary Information:**

The online version contains supplementary material available at 10.1186/s40001-023-01108-4.

## Introduction

Lower-grade glioma (LGG) is a prevalent brain tumor classified as a 2/3 grade tumor that primarily occurs in the cerebral cortex by the World Health Organization (WHO) [1]. Although considerable progress has been made in the molecular analysis and study of LGG, the treatment and survival strategies for this disease have remained stagnant over the past few decades, mainly due to its high mortality, inevitable recurrence, and significant heterogeneity [2]. In the United States, LGG accounts for approximately 43.2% of CNS gliomas, with an annual incidence of 6500–8000 new cases [3, 4]. Unlike glioblastoma (GBM, WHO grade IV, with a 5-year survival rate of 5%), LGG has an average survival time of more than 7 years but is comparatively more invasive [5, 6]. Radiation therapy has been a common treatment option for low-grade gliomas (LGGs) for many years, with proven efficacy in improving patient survival rates. However, the optimal timing, dose, and fractionation of radiotherapy for LGGs remain controversial. In the updated 2021 WHO CNS tumor classification, molecular data, namely IDH mutations in chromosomes 1p and 19q and whole arm coding deletions, now replaces the classical histology-based LGG classification. Using these molecular markers, mutations in the isocitrate dehydrogenase (IDH) 1 and IDH2 genes have been identified to classify LGG into prognostic subtypes with distinct clinical, biological, and radiological characteristics [7–10].

LGG develops through early mutations in IDH, which leads to the accumulation of 2-hydroxyglutaric acid and DNA hypermethylated phenotypes [11–13] and then acquires one among the two sets of co-existing genetic alterations: mutations in the TP53 and EGFR, or 1p/19q code deletions, and TERT, ATRX, CIC, TTN, and FUBP1 mutations [14]. Previous studies have identified a small subset of more aggressive LGGs, and these gliomas are associated with lower overall DNA methylation and pure CDKN2A/B deletions and alterations that sometimes occur at recurrence. These include temozolomide-induced hypermutation phenotypes, alterations in the Myc pathway, driver oncogenes, and tumor suppressors [15–17]. At present, LGG treatment varies by molecular subtype, stage, and location/resection and may involve clinical observation, chemotherapy (procatrazine/CCNU/vincristine or PCV and temozolomide), and radiotherapy [18]. According to clinical observations, ODG tumors respond well to chemotherapy and radiotherapy and have the best prognosis. In contrast, IDH wild-type tumors, even without high-grade histology, are linked to a poor prognosis, while IDH mutant astrocytomas are linked to a varying but moderate response [19]. In most cases, LGGs develop into higher-grade tumors, and approximately 50–75% of individuals with LGG frequently develop deterioration, pathological progression, or die. It is, therefore, crucial to find out the mechanisms of regulation of LGG initiation and progression thoroughly for biomarker identification and therapeutic targeting [20].

LGG has a wide range of genetic and phenotypic heterogeneity and is known for epigenetic alterations [21]. Processes like histone modifications, DNA methylation, chromatin remodeling, and non-coding RNAs have been the focus of attention in conventional epigenetic research [22]. Lately, various reversible chemical alterations in RNA have been suggested as a new epigenetic modality of the regulation [23]. A modification mediated by methyltransferase is N7-methylguanosine (m7G), where a CH_3_ is attached at the 7th N terminal of mRNA guanine (G). m7G modification is among the most prevailing modifications of the base during post-transcriptional modification and is primarily present at the 5’ cap of rRNA, tRNA, mRNA, and lncRNA and is essential for the maintenance of metabolism of RNA processing, stabilization, export from the nucleus and translation of proteins, including the development of tissues, formation of stem cells and their differentiation, thus controlling the heat shock response and the control of biological clock [24]. Research in recent years has highlighted that m7G modifications regulate carcinogenesis and progression, such as METTL1/WDR4-mediated aberrant translation of tRNA n7-methylguanosine modifications that promote head and neck squamous cell carcinoma-related advancement [25]. Moreover, Orellana et al. [26] revealed that these mRNAs are stabilized by an increase in the m7G modification level of a subset of tRNAs by the METTL1/WDR4 complex. It also improves the efficiency of their translation, prevents them from getting decayed, reduces ribosomal arrest, is correlated with low survival in cancer, and directly translates [27]. However, m7G methylation modifications’ specific role in LGG is unclear.

Moreover, the tumor immune microenvironment is remarkably involved in the invasion of the tumor. During the growth of the tumor, it interacts with its microenvironment through cell signaling by means of infiltrating immune cells or molecular mechanisms. LGG can activate many types of immune cells, such as tumor-infiltrating macrophages, which secrete cytokines in large quantities along with growth factors and interleukins, resulting in an appropriate tumor microenvironment (TME) that promotes the development and proliferation of glioma cells [28]. Through blocking signaling pathways, including programmed cell death protein 1/PD ligand 1(PD-1/PD-L1), which inhibits the function and response of infiltrating immune cells, tumor cells cause immune escape. Additionally, the surplus amount of sugar and amino acids are utilized in tumor cells’ metabolic reconstruction, depriving the T cells of their nutritional needs, inactivating them, and suppressing immune response [29]. Furthermore, one of the main mechanisms for the development of immunosuppressive TME is immunosuppressive cell recruitment and expansion in TME, including T regulatory lymphocytes, tumor-associated macrophages, and myeloid-derived suppressor cells (MDSCs) [30]. Similarly, combinations of anticancer and multitargeted immunotherapeutic agents can prevent adaptive resistance and remarkably enhance the prognosis of tumors and survival [31]. Therefore, accurate diagnosis and effective treatment of glioma depend on a thorough understanding of key molecules and processes.

In the current research, we assessed the expression profile of m7G regulators in gliomas. Bioinformatics analysis was carried out on a large scale by retrieving gene expression data from commonly used databases. Afterward, the expression of critical genes in m7G regulators in specimens of the tumor and healthy brain tissue was validated by means of western-blotting, real-time quantitative polymerase chain reaction (qPCR), as well as immunohistochemistry (IHC). Moreover, we systematically and comprehensively assessed the m7G score prognostic value in glioma treatment. Considerable differences (variations) in m7G score expression among glioma patients of different ages and genders were observed, and a major m7G score expression tendency in various types of mutations was discovered. Furthermore, we assessed the relationship of the m7G score with immune infiltration level. It was noted that m7G Score expression was significantly upregulated in infiltration with TME cells. Moreover, by qPCR, western-blotting, and IHC, we detected that the m7G Score hub gene was expressed at different levels in normal brain tissue, refractory epilepsy tissue, and lower-grade gliomas. According to these data, it is concluded that the m7G score is a potential prognosis biomarker, and it can possibly be a clinical therapeutic target for individuals suffering from glioma.

## Methods and materials

### Data source and preprocessing of lower-grade glioma

The study utilized RNA-Seq data from the TCGA database, which contained 491 LGG samples with WHO classification II-III, and 103 normal cortical samples from the GTEx project, which served as normal sample controls. To ensure the accuracy of subsequent modeling, 481 samples with duplicate sequencing, unclear WHO classification, non-primary LGG, overall survival time less than 1 day, and no survival status were excluded. Furthermore, external validation was conducted using samples with complete survival information from the CGGA-693 project (332 patients) and the CGGA325 project (162 patients). As the CGGA and TCGA data were generated using RNA sequencing on the Illumina platform, the data underwent background correction, normalization, and expression calculation with the combat function in the sva package. The data were log(x + 1) normalized and transformed to TPM to remove batch effects. The study also obtained data related to somatic mutations and CNV from the TCGA-LGG cohort, which included 505 samples. The Meta cohort was created by integrating data from the TCGA-LGG, CGGA-693, and CGGA-325 cohorts. M7G-linked genes were retrieved from existing literature [24], and the sets of related gene GOMF_M7G_5_PPPN_DIPHOSPHATASE_ACTIVITY, GOMF_RNA_CAP_BINDING, and GOMF_RNA_7_METHYLGUANOSINE_CAP_BINDING were also obtained.

### Unsupervised clustering of m7G modification pattern

Unsupervised consistent clustering analysis was carried out on the basis of the m7G regulators or m7G patterns regulating gene expression levels. To ascertain if each isoform was relatively independent of the other isoforms, principal component analysis (PCA) was utilized. The R software “conensusClusterPlus” was utilized to find out the number of clusters, and for verification of the stability of subtypes, 1000 replicates with pltem equal to 0.8 were carried out.

### Identification of the m7G methylation regulator risk score in lower-grade glioma

Initially, we performed a prognostic analysis of DEGs extracted from different m7G clusters, and we screened genes at *P* < 0.05 for every overlapping DEG using Cox regression methods. The principal components 1 and 2 were both chosen as feature scores, m7G score = ∑ (PC1i + PC2i).

### Biological enrichment analysis for distinct m7G modification patterns

We used GSVA to determine differences in terms of biological pathways across subtypes. For the annotation of genes' biological activities, molecular mechanisms, and cellular components, Gene Ontology (GO) was utilized. We used the Kyoto Encyclopedia of Genes and Genomes (KEGG) to annotate gene pathways. The limma package was utilized to observe the differentially expressed genes across the isoforms (*p* < 0.05), and after identifying DEGs, GO and KEGG analysis was performed employing the clusterProfiler package. Furthermore, c2.cp.kegg.v7.0.symbols.gmt was utilized as the reference gene set with screening threshold set at FDR < 0.05. Activation of classical cancer pathways was calculated using 11 representative basal pathways from the h.all.v7.4.symbols gene set.

### Analysis of drug sensitivity

IC50 was measured employing the R package “prophetic,” and chemotherapeutic medications were retrieved from the genome of the Drug Sensitivity in Cancer (GDSC).

### Evaluating TME and immune cells infiltration in lower-grade glioma

For immune cell analysis, to evaluate immune cell abundance across different samples, we employed various algorithms such as TIMER, CIBERSORT, QUANTISEQ, MCP-counter, XCELL, and EPIC simultaneously. Furthermore, the ESTIMATE algorithm was utilized to measure the immune score and mesenchymal score to find out the microenvironmental status.

### Developing protein–protein interaction (PPI) network and identification of hub genes

To elucidate the molecular mechanisms of LGG further, a DEG interaction network was developed employing the STRING database (https://string-db.org/). The relationships among DEGs were subsequently assessed using Cytoscape v3.8.2 software. The Cytoscape plug-in application Molecular Complex Detection (“MCODE”) was also utilized to re-analyze clusters in the network following the given criteria: degree cutoff = 2, node score cutoff = 0.2, k-core = 2, and maximum depth = 100. The top 5 central genes were filtered as m7G-related hub genes using the Cytoscape plug-in “cytoHubba.”

### Molecular docking

Virtual screening of m7G-related Hub gene molecular docking was done using AutoDock Vina 1.1.2 to predict the most likely best ligand. Constituent structures were obtained at Pubchem. Molecular optimization was performed using SYBYL-X software with the following optimization parameters set: The energy gradient was restricted to 0.005 kcal/(mol-A), the Tripos force field was employed, the Gasteiger-Hückel charge was chosen, the maximum iteration factor was adjusted to 10,000, and all other parameters were set at their default settings. We downloaded the target structure at RCSB (https://www1.rcsb.org/). Through the Surflex-Dock module of SYBYL-X, the crystalline water of the protein was removed, and the terminal residues were processed. Furthermore, the ligands were extracted, hydrogenated, and energy-optimized to produce a binding pocket followed by molecular docking.

### Extraction of RNA and qRT-PCR

Between January 2022 and June 2022, ten lower-grade glioma tissues and ten healthy brain tissues were obtained from 20 individuals who went through surgical dissection and pathological confirmation at the First Affiliated Hospital of Xinjiang Medical University. Approval for this research was granted by the Medical Research Ethics Committee of the same hospital. Total RNA was extracted by RNA reagent (Servicebio), and the concentration of total RNA was measured utilizing NanoDrop2000 (Thermo Fisher Scientific, United States). A two-step reaction process, reverse transcription (RT) and polymerase chain reaction (PCR) were carried out to determine levels of mRNA. In addition, cDNA synthesis was carried out utilizing the Servicebio RT First Strand cDNA Synthesis Kit (Wuhan servicebio Technology CO., LTD, China). Expression levels of GAPDH, EIF4E, EIF4E3, EIF4E2, NCBP1, and NCBP2 were determined by qRT-PCR with the aid of SYBR Green qPCR Master Mix (High ROX) (Servicebio, Wuhan, China) to detect them. The results were expressed as GAPDH. Designing and synthesis of PCR primer sequences were done by Servicebio (Wuhan) Co: GAPDH-F:5′- GGAAGCTTGTCATCAATGGAAATC-3′,GAPDH-R:5′- TGATGACCCTTTTGGCTCCC -3′, EIF4E -F: 5′- CGGAATCTAATCAGGAGGTTGCT -3′, EIF4E -R: 5′- CTCATCTTCCCACATAGGCTCAA -3′, EIF4E3 -F: 5′- GTGGCGTATGGAAGATGAAAGTC -3′, EIF4E3 -R: 5′- TCCCGAACACTGACACTAACTCC -3′, NCBP1 -F: 5- GACCTTATCTTGCCTTTGACAGC -3′, NCBP1 -R: 5- CCTTCCAGTGGGACTTAATGATG -3′. NCBP2 -F: 5′- GCTTTAAGGAGGGCAGGCAATA -3′, NCBP2 -R: 5′- CATAGCCTCCTCTCCCAGCATC -3′, EIF4E2-F: 5′- TGTGGAGCAGTTCTGGAGGTT-3′, EIF4E2-R: 5′- CGAATAATCCACTTGCCACCA -3′. The relative expression levels of EIF4E, EIF4E3, EIF4E2, NCBP1, and NCBP2 were quantified by the 2(^−ΔΔCT^) formula.

The amplification reaction was as follows: pre-denaturation at 95 °C for 10 min, subsequent denaturation at 95 °C for 40 cycles of 15 secs, and extension at 60 °C for 30 secs. We also recorded fluorescence signals from 65 to 95 °C at an interval of 0.3 °C.

### Western blot analysis and antibodies

RIPA buffer (Servicebio, Wuhan) was employed for total cellular protein lysis. Following ultrasonic cracking, the quantification of lysates was done by a BCA Protein Assay kit (Servicebio, Wuhan). 10% SDS‐PAGE gel was utilized for the separation of total protein, which was transferred to a PVDF membrane (Servicebio, Wuhan). Following the incubation of antibodies, a chemiluminescence system (CLINX, Shanghai) was utilized for the detection of signals. The primary antibodies (all with the same dilution ratio of 1:1000 provided by ProteinTech Group, Wuhan, China) we utilized were anti‐rabbit NCBP1(catalog no. 10349-1-AP), NCBP2(catalog no. 11950-1-AP), EIF4E(catalog no. 11149-1-AP), EIF4E3(catalog no. 17282-1-AP), EIF4E2(catalog no. 12227-1-AP), and anti‐mouse GAPDH(dilution ratio of 1:2000, Servicebio, catalog no. GB15002). The anti‐rabbit and anti‐mouse (both with a dilution ratio of 1:5000, catalog nos. GB23303 and GB25301, respectively, Wuhan, China) secondary antibodies were purchased from Servicebio. GAPDH was employed as an internal control.

### Immunohistochemistry and hematoxylin–eosin staining

The m7G scores hub genes were validated experimentally by immunohistochemistry (IHC) staining. Ten samples of LGG tissues and ten intractable epilepsy tissue samples were retrieved from individuals at the First Affiliated Hospital of Xinjiang Medical University. Prior to the retrieval of tissue samples, none of the individuals underwent anti-cancer therapy. An informed consent form was signed by each patient, and the hospital's ethics committee granted its approval.

Immunohistochemistry (IHC) staining was performed after tissues were fixed and paraffinized. Slides were sliced to a 5 μm width, dewaxed, and rehydrated. H2O2(3%) was used for 10 min to block endogenous peroxidase after incubation for 1 h at room temperature with a 3% blocking solution of bovine serum albumin(BSA). To inhibit endogenous peroxidases, hydrogen peroxide was administered to the cells for 10 min. The relevant protein antibodies were used to incubate the sections at 4 °C overnight. The antibodies used were as follows: NCBP1 (Cat. No. 10349-1-AP, 1:1000), NCBP2 (Cat. No. 11950-1-AP, 1:1000), EIF4E (Cat. No. 11149-1-AP, 1:1000), EIF4E3 (Cat. No. 17282-1-AP, 1:1000) and EIF4E2 (Cat. No. 12227-1-AP, 1:1000) antibodies were acquired from Proteintech (Wuhan, China); Subsequently, the incubation of the sections was done with a biotinylated goat anti-rabbit secondary antibody for a half-hour at 37 °C (Cat. No. GB23383, 1:200, Servicebio, Wuhan, China). A freshly synthesized 3,3′-diaminobenzidine(DAB) reagent was used for color development(Boster, Wuhan, China). Each tissue segment underwent independent IHC staining by two pathologists.

### Statistical analysis

Correlation coefficients between immune cells and m7G regulator expression were computed by Spearman correlation analysis. The Kruskal–Wallis test was employed for variations between the three groups, and the *x*^2^ test was utilized for associations between categorical covariates. As per the correlation between the m7G score and the prognosis of affected individuals, the optimal cutoff value for the individual data set subgroup was determined with the aid of the survminerR package. Individuals were classified into high and low m7Gscore subgroups on the basis of this value. The log-rank statistic was employed to reduce the batch effect of the calculation. The Kaplan–Meier method was employed for plotting OS, and for identifying statistical variations, a log-rank test was employed. (restricted to 10 years of follow-up). Univariate Cox regression was utilized to calculate risk ratios for m7G modifiers and genes linked to the m7G phenotype. Independent survival factors were identified by conducting multivariate Cox regression, and the Maftools and its “oncoplot” function were utilized to visualize mutational variations. In addition, by employing Gene Expression Profile Interaction Analysis (GEPIA) (http://gepia.cancer-pku.cn/), further differential expression analysis was carried out on LGG samples from TCGA (*N* = 518) and healthy samples (*N* = 207) from matched TCGA normal and genotype-tissue expression (GTEx) data. The value of *p* < 0.05 was considered statistically significant.

## Results

### Landscape of m7G regulators in LGG

**Fig. 1:**
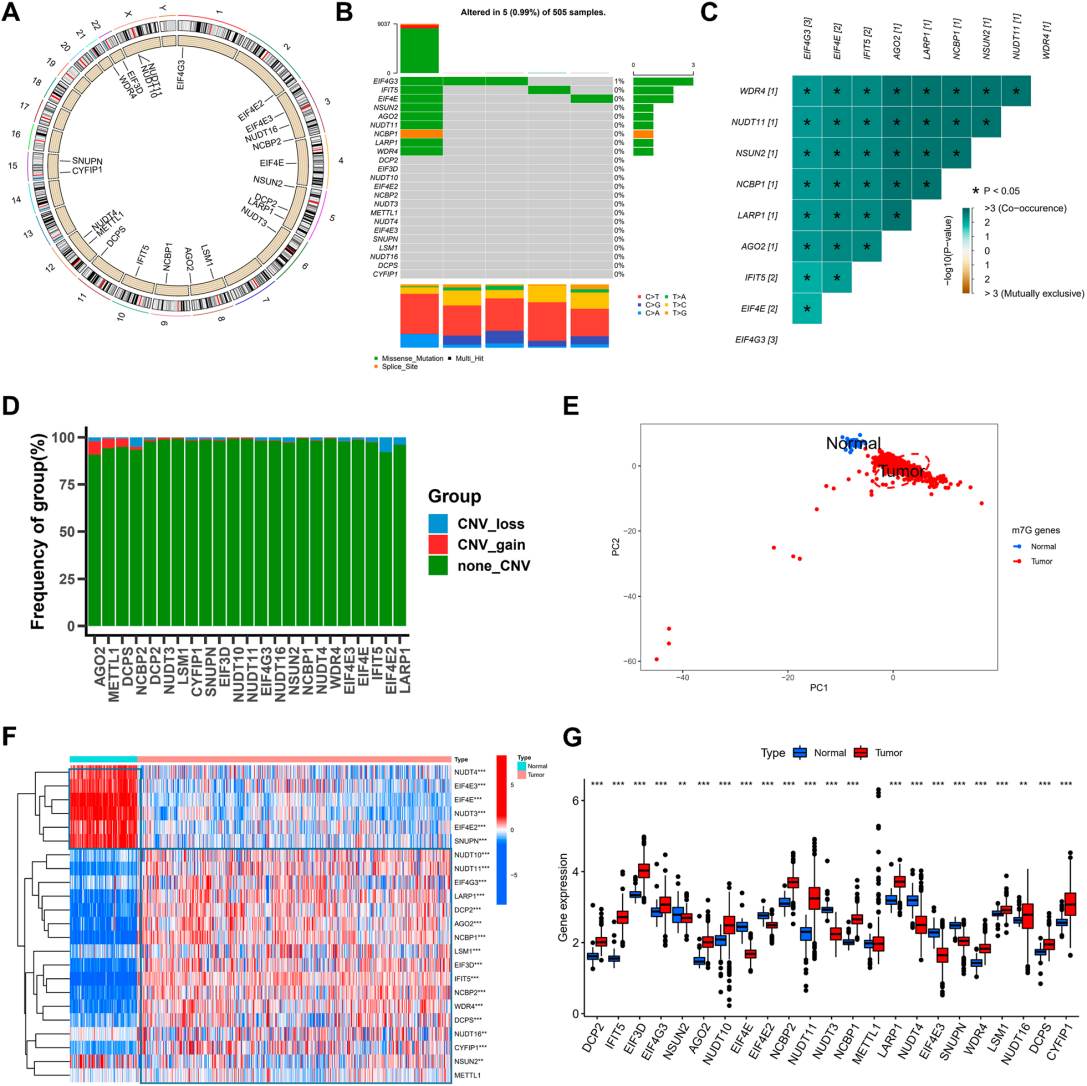
Landscape of m7G regulators in LGG. **A** The 23 chromosomal regions where m7G regulator CNV alterations were found in TCGA-LGG cohorts. **B** The frequency of m7G regulator mutations and their categorization. **C** Using Pearson correlation analysis, a correlation plot drawn between the top 9 m7G regulator mutation frequencies. *p* < 0.05 was considered significant. **D** The m7G regulators’ CNV variation frequency in the TCGA-LGG cohort. **E** The m7G regulators-transcriptome profiles of healthy and malignant tissue were analyzed using principal component analysis, which revealed a remarkable difference between the various samples. **F** The m7G regulators’ expression varied between healthy and malignant tissues, as shown by a heatmap. **G** The 23 m7G regulators’ expression in LGG tissue from the TCGA-LGG cohort versus healthy tissues from Genotype-Tissue Expression samples. The Kruskal–Wallis test was used to compare the difference. * *p* < 0.05; ** *p* < 0.01; *** *p* < 0.001

In this study, 23 m7G-related genes were retrieved from the TCGA cohort, and their locations on chromosomes are illustrated in Fig. 1A. Initially, the copy number variation was summarized, and the incidence of somatic mutations in genes linked to m7G in LGG. Only 5 of the total 505 samples were mutated with a frequency of 0.99%, with EIF4G3 exhibiting the highest mutation frequency (Fig. 1B), and further analysis revealed that EIF4G3 and EIF4E, IFIT5, AGO2, LARP1, NCBP1, NSUN2, NUDT11, and WRD4 (Fig. 1C). CNV alteration frequency showed that CNV alterations were prevalent in m7G-related genes, AGO2 was concentrated in copy number expansion, while the frequency of CNV deletion was common in EIF4E2 (Fig. 1D). Upon combining specimens of LGG from the TCGA database and healthy cortical samples from the GTEx database, it was found that LGG samples could be distinguished entirely from the healthy ones as per the expression of genes related to m7G (Fig. 1E). To confirm if the above-mentioned mutants affect the expression of genes related to m7G- in individuals with LGG, we obtained the mRNA expression of m7G-related genes in healthy and LGG samples, and all the genes linked to m7G were remarkably differentially expressed across samples (Fig. 1F, G). NUDT4, EIF4E3, EIF4E, NUDT3, EIF4E2, and SNUPN showed higher expression in normal tissues compared to LGG samples. The above analysis showed that m7G-related genes were highly heterogeneous between samples of healthy and LGG tissues, indicating that imbalanced expression of genes related to m7G is crucially involved in the onset and progression of LGG.

### Modification patterns mediated by m7G-regulators in lower-grade glioma

**Fig. 2:**
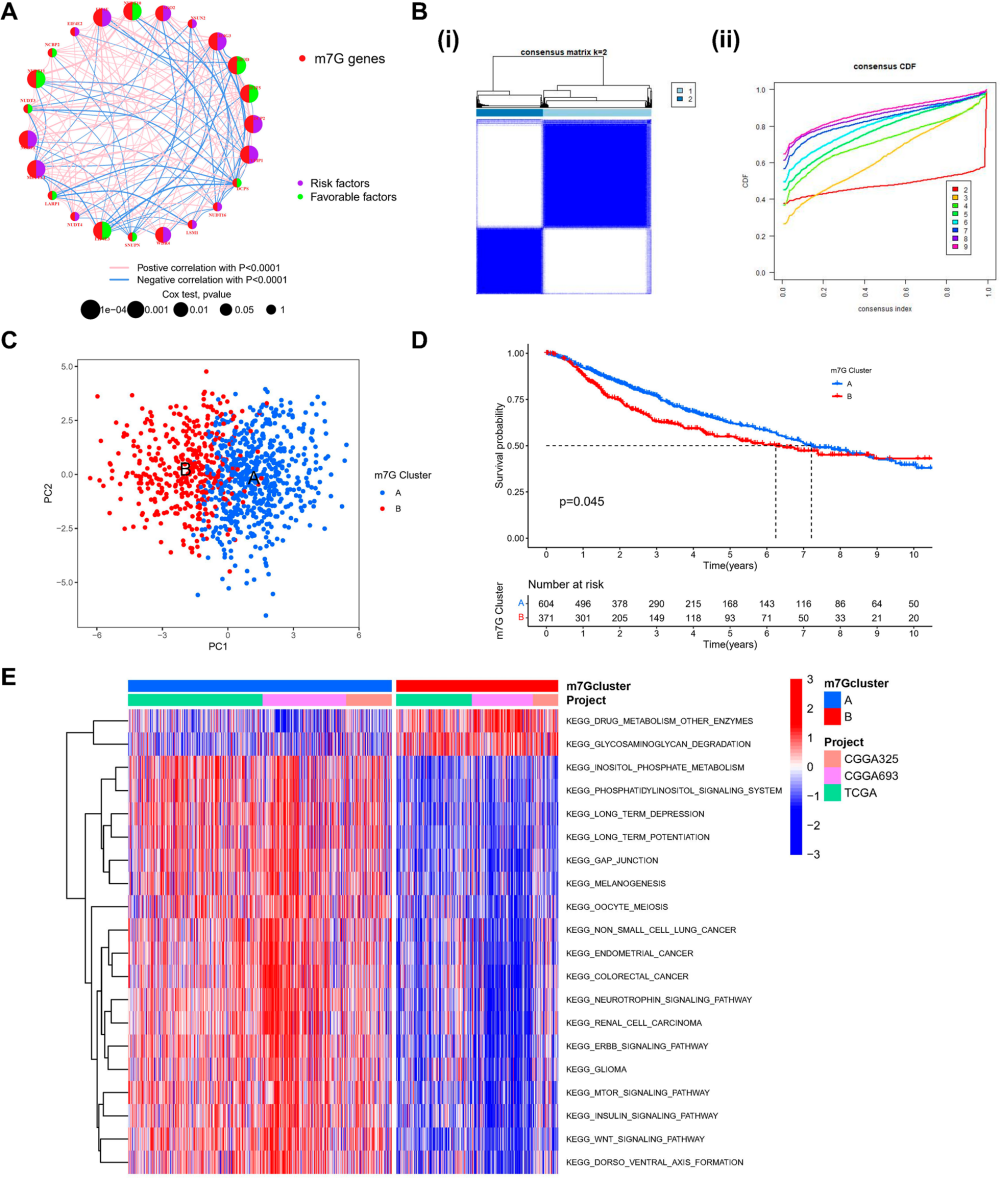
Identification of m7G methylation modification profiles. **A** The m7G regulators interactions in LGG. The diameter of the circle indicated the significance level of the *P* values obtained from the Log-rank test, which were, successively, *p* < 1e-04, p < 0.001, *p* < 0.01, *p* < 0.05, and *p* < 1. Risk factors are denoted in purple, whereas favorable factors for overall survival are denoted in green. The connecting lines show relationships of m7G regulators as determined by Spearman correlation analysis. Pink represents a positive correlation and blue represents a negative correlation. **B** (i) The consensus matrix for *k* = 2 produced using consensus clustering is shown by the heatmap. (ii) Relative change in consensus CDF area under the curve for *k* = 2 to 9. **C** Two m7G modification subtypes’ transcriptome profiles underwent principal component analysis, revealing a significant variation in modification patterns. **D** Kaplan–Meier curves with Log-rank p values < 0.05 demonstrated a significant variation in survival between the two m7G modification patterns. These analyses were based on the TCGA-LGG, CGGA-325, and CGGA-693 cohort, which included 604 patients in m7Gcluster-A and 371 cases in m7Gcluster-B. **E** GSVA of biological pathways between two distinct subgroups

Three datasets with complete prognostic information (TCGA-LGG, CGGA-693, CGGA-325) were included in a Meta cohort of 975 LGG patients. In the Kaplan–Meier survival analysis and log-rank test, the value of prognosis in 20 m7G-associated genes for LGG patients was revealed, distinguished by the best cut-off value in each group (Additional file 1: Fig S1). m7G-associated gene network specifically described the combined gene interactions and their prognostic value for patients having these genes (Fig. 2A). It was discovered that the majority of the m7G-related genes showed significant correlations and had a better prognostic indication. The aforementioned outcomes indicate that crosstalk between m7G-related genes might be crucially involved in forming different m7G methylation modification patterns. The consensusClusterPlus software was employed to categorize subjects on the basis of the expression of prognostic genes linked to m7G, and when the *K* value = 2, the CDF downward slope was minimal (Fig. 2B), and two different modification patterns were finally identified; pattern A with 604 cases and pattern B with 371 cases. These patterns were defined as m7G clusters. PCA analysis revealed that the two patterns have a relatively discrete nature (Fig. 2C). Predictive analysis revealed a significant survival advantage in the m7 Gcluster-A modification pattern (Fig. 2D). To explore the physiological activities between these different m7G modification patterns, we carried out a GSVA enrichment analysis. As illustrated in Fig. 2E, compared with m7Gcluster-B, m7Gcluster-A presented a significant survival advantage with inositol phosphate metabolism, phosphatidylinositol, glioma, neurotrophin, ErbB, mTOR, insulin, Wnt signaling pathways as well as other enrichment pathways. The above results may provide evidence to support the prognostic advantage of m7G cluster-A.

### TME cell infiltration in various m7G modification patterns

**Fig. 3:**
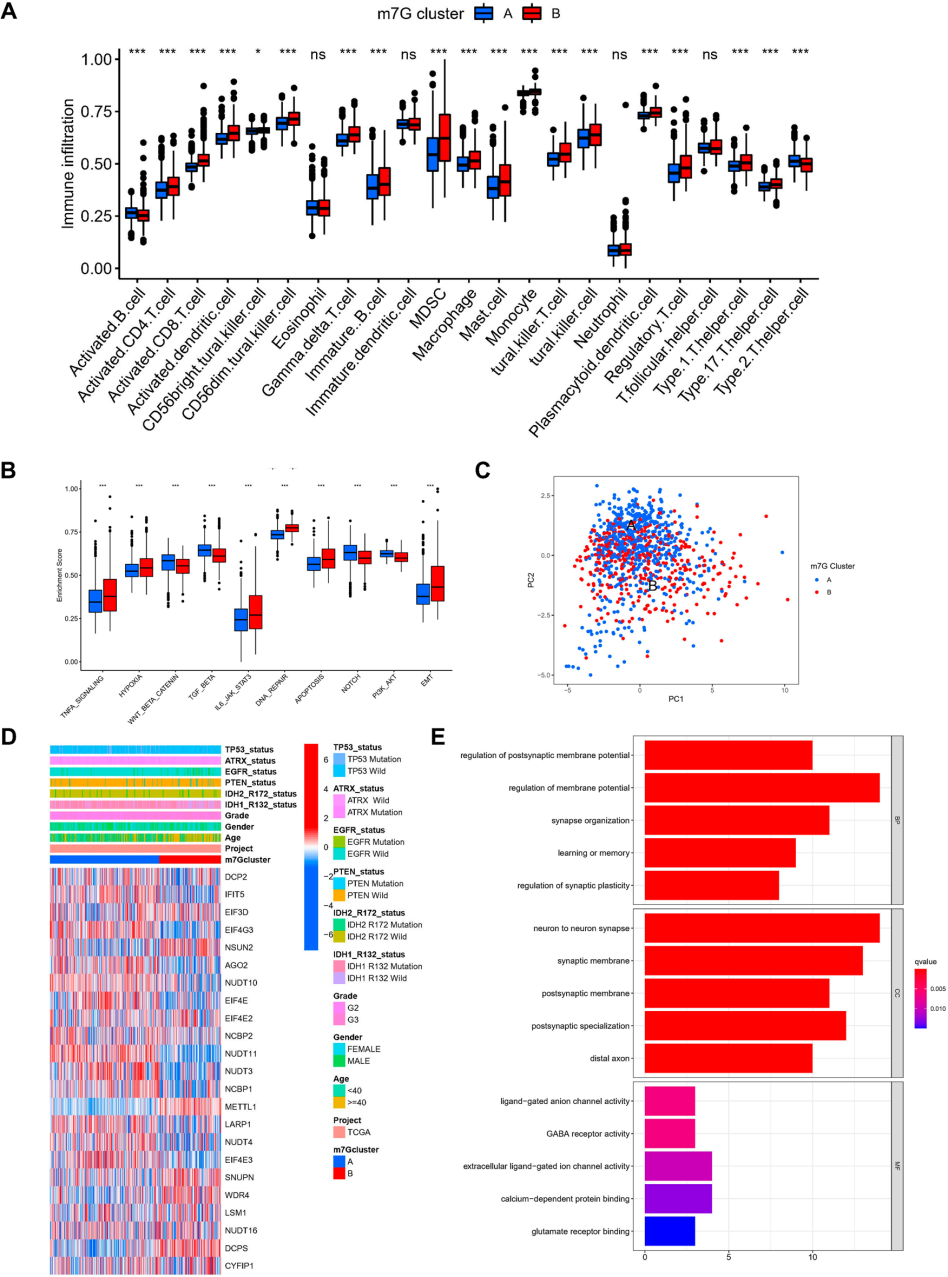
Distinct immune microenvironments in m7G modification patterns and each pattern's biological features. **A** 23 immune cells' differential expression between two m7G modification patterns. **B** Differential expression of two m7G modification patterns’ Hallmark pathway features. *** *p* < 0.001, ** *p* < 0.01, * *p* < 0.05. **C** PCA scatter plot for the methylation modification pattern of the m7G gene. **D** Heat map of the distribution of clinicopathological characteristics between two m7G modification patterns. **E** GO enrichment analysis was utilized to functionally annotate the genes associated with m7G on the bar chart

The analysis of TME surprisingly highlighted that m7G cluster-B was abundant in immune cell infiltrates, including macrophages, eosinophils, natural killer cells, mast cells, MDSC, and dendritic cells (Fig. 3A). Subsequent analysis showed significantly enhanced hallmark pathway activity in m7Gcluster-B, such as activation of HYPOXIA, EMT, and other pathways (Fig. 3B). Although there was partial overlap, PCA analysis likewise showed a potential distinction between the two m7G modification patterns based on cancer activity pathway scores (Fig. 3C). In addition, in a separate TCGA cohort, we likewise found significant upregulation of most of the m7G genes in the m7G cluster-A and a significantly different distribution of clinical traits (Fig. 3D).

### Exploration of m7G modification pattern regulatory genes and related molecular isoforms

**Fig. 4:**
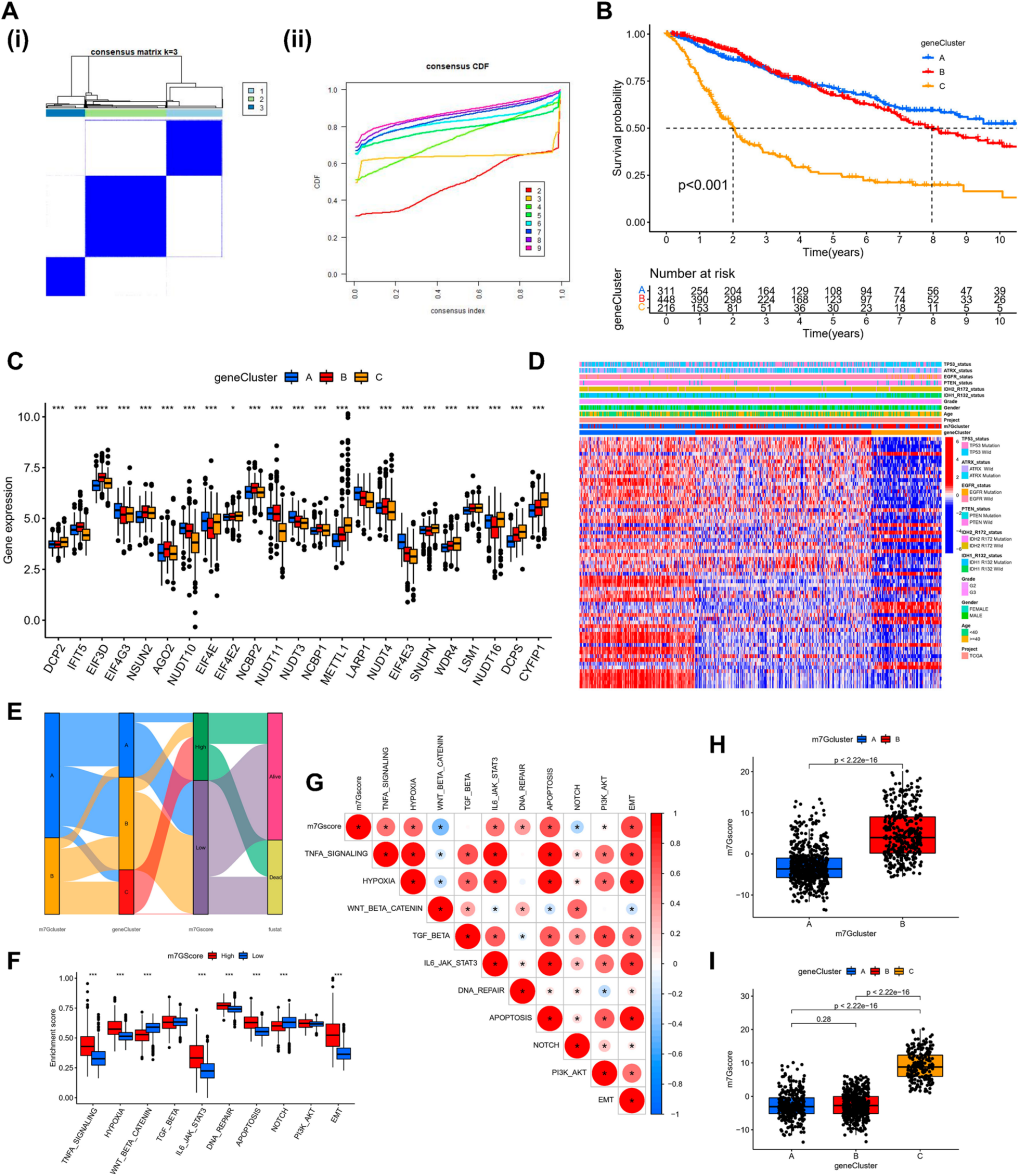
m7G scores and phenotypic identification of genes linked to m7G. **A** (i) The consensus matrix for *k* = 3 generated by means of consensus clustering is shown by the heatmap (ii) Relative change in consensus CDF area under the curve for *k* = 2 to 9. **B** Various gene cluster-related survival curves (p < 0.001, Log-rank test). **C** The differential expression of genes linked to m7G among several gene clusters. *** *p* < 0.001, ** *p* < 0.01, * *p* < 0.05. To examine the statistical variations across the three gene clusters, the one-way ANOVA test was applied. **D** Heat map of genetic modification patterns. **E** Sankey diagrams illustrating several genotypes. **F** Differential expression of Hallmark pathway characteristics between high-m7G score and low-m7G score. *  *  * *p* < 0.001. **G** Relationship between immune cells and m7G scores, with red denoting a positive correlation and blue denoting an inverse correlation. **H** m7G score’s differential expression within the m7G cluster (*p* < 0.001). **I** m7G score’s difference analysis in the gene cluster (Kruskal–Wallis test)

To assess the possible biological functions of each m7G modification pattern in further detail, the limma package was employed to identify 70 differentially expressed genes (DEGs) related to the m7G phenotype. The clusterProfiler package was utilized to carry out the GO enrichment analysis on the DEGs, which, surprisingly, showed an association with the postsynaptic membrane potential regulation, membrane potential regulation, synapse organization, neuron to neuron synapse, synaptic membrane, ligand-gated anion channel activity, and other related biological processes were enriched (Fig. 3E). To confirm this regulatory mechanism in a thorough manner, we carried out an unsupervised clustering analysis according to the collected prognosis-related m7G modification pattern-related genes to classify subjects into various genetic subtypes. Similar to the grouping of m7G modification patterns, the unsupervised clustering algorithm highlighted three different genetic subtypes, called gene clusters-A, B, and C (Fig. 4A). 311 of the 975 LGG patients clustered in genotype A, 448 in genotype B, and 216 in genotype C, but with a poorer prognosis (Fig. 4B). The prognosis was poor (Fig. 4B). Major variations in the m7G regulators’ expression were observed in the aforementioned genetic subtypes, which is similar to the expected outcomes of m7G methylation modification patterns, along with significant upregulation of m7G-related genes in their subtype A (Fig. 4C, D).

### Identification of m7G-regulators genes’ phenotypes and m7G scores

The above analysis is based only on the population of patients and cannot predict the pattern of m7G methylation modifications with accuracy in each individual. Taking the individual heterogeneity of m7G modifications into consideration, in accordance with these phenotype-related genes, we established a PCA algorithm-based score to systematically quantify the pattern of m7G modifications in LGG patients, which we called the m7G score. We divided the cut-off value of the derived m7G score into two groups, high and low, for all LGG patients (cut-off = 1.530208). Mulberry plots showed the association between the subtypes (Fig. 4E). The ssGSEA algorithm analysis highlighted that the hallmark pathway activity was considerably enhanced in high-score patients (Fig. 4F), while analysis of the associated pathway activity highlighted that high scores were considerably linked to increased activation of the pathway (Fig. 4G). Kruskal–Wallis test showed that m7G clusters were significantly different from each other, with the highest score in m7G cluster-B (Fig. 4H); similarly, m7G scores were significantly different between gene clusters, with the highest score in gene cluster-C (Fig. 4I). Therefore, the above outcomes strongly suggest that the m7G Score can better assess individual patients’ m7G modification patterns.

### Prognostic value of m7G scores in individual LGG patients

**Fig. 5:**
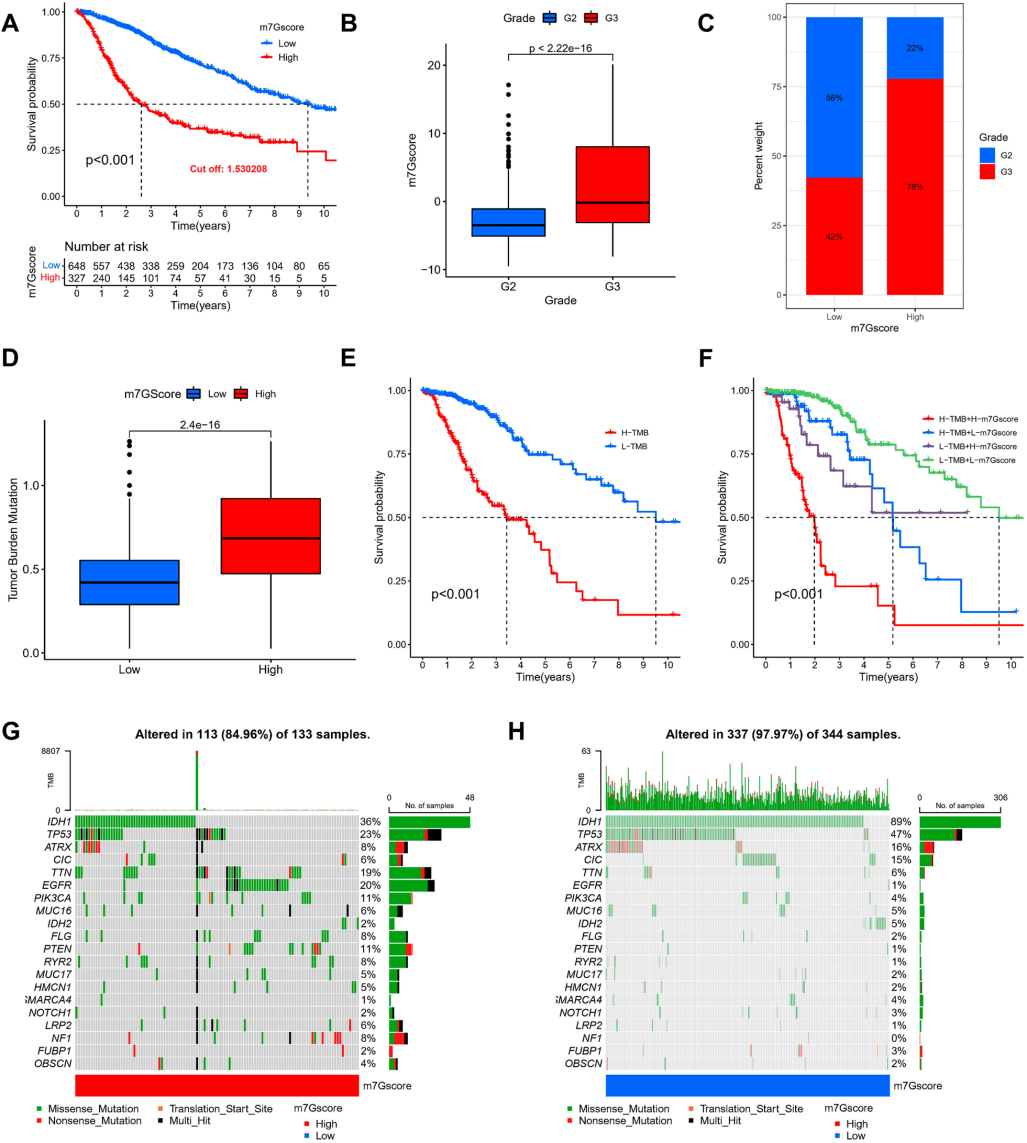
Assessment of clinical prognosis of m7G scores and somatic tumor mutations. **A** Survival analysis of high- and low-m7G score groups using Kaplan–Meier curves (*p* < 0.001, Log-rank test). **B** Comparison of the m7G score among the individuals of Grade 2 and Grade 3 groups (*p* < 0.001, Wilcoxon test). **C** The proportion of Grade 2 and Grade 3 in high- and low-m7G score groups. **D** Stratified analysis of the m7G score for individuals with LGG by tumor mutation burden (*p* < 0.001, Wilcoxon test). **E** Survival analysis of TMB (*p* < 0.001, Log-rank test). **F** Survival analysis of TMB along with m7G score (*p* < 0.001, Log-rank test). **G** Waterfall chart of the high-m7G score group. **H** Waterfall chart of the low-m7G score group

We further determined the significance of the m7G score in predicting the prognosis of affected individuals. Subjects with lowered m7G scores showed significant survival benefits (Fig. 5A), and the more advanced the patients, the higher the m7G score (Fig. 5B, C). In addition, we found that the m7G score also had a good survival differentiation value in different clinical subgroups, such as different age subgroups (Additional file 2: Fig S2A) and gender subgroups (Additional file 2: Fig S2B). In addition, in the classical oncogenic mutation status (TP53, EGFR), the m7G score also had a better prognostic indication value (Additional file 2: Fig S2C). TMB has an essential role in guiding immunotherapy-based schemes in LGG patients, and given the clinical importance of TMB, we tried to explore the intrinsic correlation between TMB and m7G score. It was found that the lower TMB score in the Low m7G Score group (Fig. 5D) and the Low-TMB grouping represented a better prognostic outcome (Fig. 5E), and when the Low m7G score was combined with Low-TMB indicated a better prognostic outcome (Fig. 5F). Furthermore, in both TCGA (Additional file 4: Fig S4A) and CGGA cohorts (Additional file 4: Fig S4B), m7G Score was considered to be an independent risk factor, and the risk distribution status plots demonstrated the distribution of patients, m7G Score scores, and change in survival status in both groups (Additional file 4: Fig S4C), and a decrease in OS with increasing m7G Score (Additional file 4: Fig S4D). We then analyzed the difference in somatic mutation distribution in the low and high m7G score in the TCGA-LGG cohort by means of the maftools package, where IDH1 was the most widely mutated gene in the two groups, while the TTN mutation rate was 36% in the high m7G Score group (Fig. 5G) compared to 89% in the low m7G Score group (Fig. 5H).

### The function of m7G fraction in anti-PD-1/PD-L1 immunotherapy

**Fig. 6:**
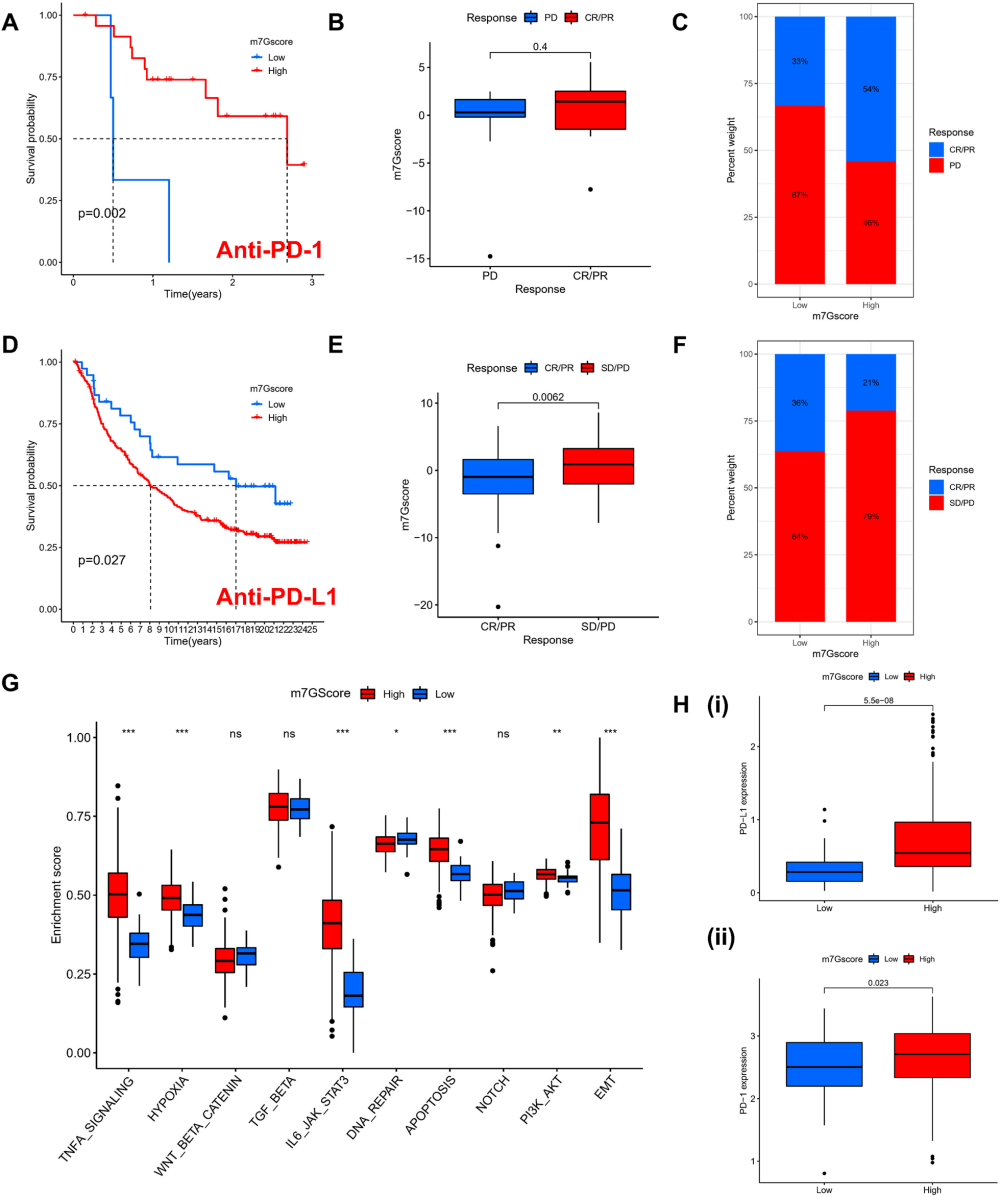
The assessment of immunotherapy response by the m7G score signature in the Anti-PD-1 and Anti-PD-L1 cohort. **A** The Kaplan–Meier curve analysis of high- and low-risk m7G score in the Anti-PD-1 cohort. **B** The comparison of the m7G score between individuals making complete/partial response (CR/PR) and those who kept a stable/progressive disease (SD/PD). **C** The proportion of individuals with LGG made complete/partial response (CR/PR) or kept a stable/progressive disease (SD/PD) in high- and low-risk m7G scores. **D** The Kaplan–Meier curve analysis between high- and low-risk m7G score in the Anti-PD-L1 cohort. **E** Comparing m7G scores between subjects making CR/PR and the subjects who kept an SD/PD in the Anti-PD-L1 cohort. **F** The proportion of individuals with LGG made CR/PR or kept an SD/PD in high- and low-risk m7G score of the Anti-PD-L1 cohort

Individuals exhibiting a high TMB status had long-lasting clinical responses to anti-PD-1/PD-L1 immunotherapy. Therefore, the aforementioned findings indirectly demonstrate that the differential modification pattern of tumor m7G might be a key factor in regulating the clinical response to PD-1/PD-L1 immunotherapy. The PD-L1 and PD-1 blockade-represented immunotherapy has clearly become a significant advancement in cancer treatment. This research checked if M7G modification characteristics could predict the response of individuals to immune checkpoint blockade therapy according to two immunotherapy cohorts: GSE78220 for the anti-PD-1 immunotherapy cohort and IMvigor210 for the anti-PD-L1 immunotherapy cohort. Individuals with high m7G Scores in the anti-PD-1 cohort demonstrated major clinical benefit and prolonged survival (Fig. 6A). However, no remarkable variation was seen in immunotherapy outcomes among individuals belonging to various m7G score subgroups (Fig. 6 B–D). In the anti-PD-L1 cohort, individuals with low m7G scores had better prognostic outcomes (Fig. 6D) and were more inclined to CR/PR (Fig. 6E, F). The aforementioned observation implies that the quantification of m7G modification patterns can serve as a potential and viable biological marker for analyzing the prognostic and clinical response of individuals to immunotherapy and that different statuses of m7G modification may represent the response to PD-1 or PD-L1, but the mechanism is unknown. Subsequently, we predicted the level of activity of their hallmark pathway in an anti-PD-L1 immunotherapy cohort and found that, like the m7G Cluster, individuals with a high m7G score had more significant activation (Fig. 6G). In addition, the expression levels of PD-L1 and PD-1 were also considerably upregulated in patients with high m7G scores (Fig. 6H). In conclusion, this research clearly indicates that m7G methylation modification patterns were correlated remarkably with tumor immunophenotype and response to anti-PD-1/L1 immunotherapy and that the developed m7G modification profile will help predict response to anti-PD-1/L1 immunotherapy as well as in PD-1/L1 immunotherapy.

### Identification of the indicative role of m7G scores in the immune microenvironment

**Fig. 7:**
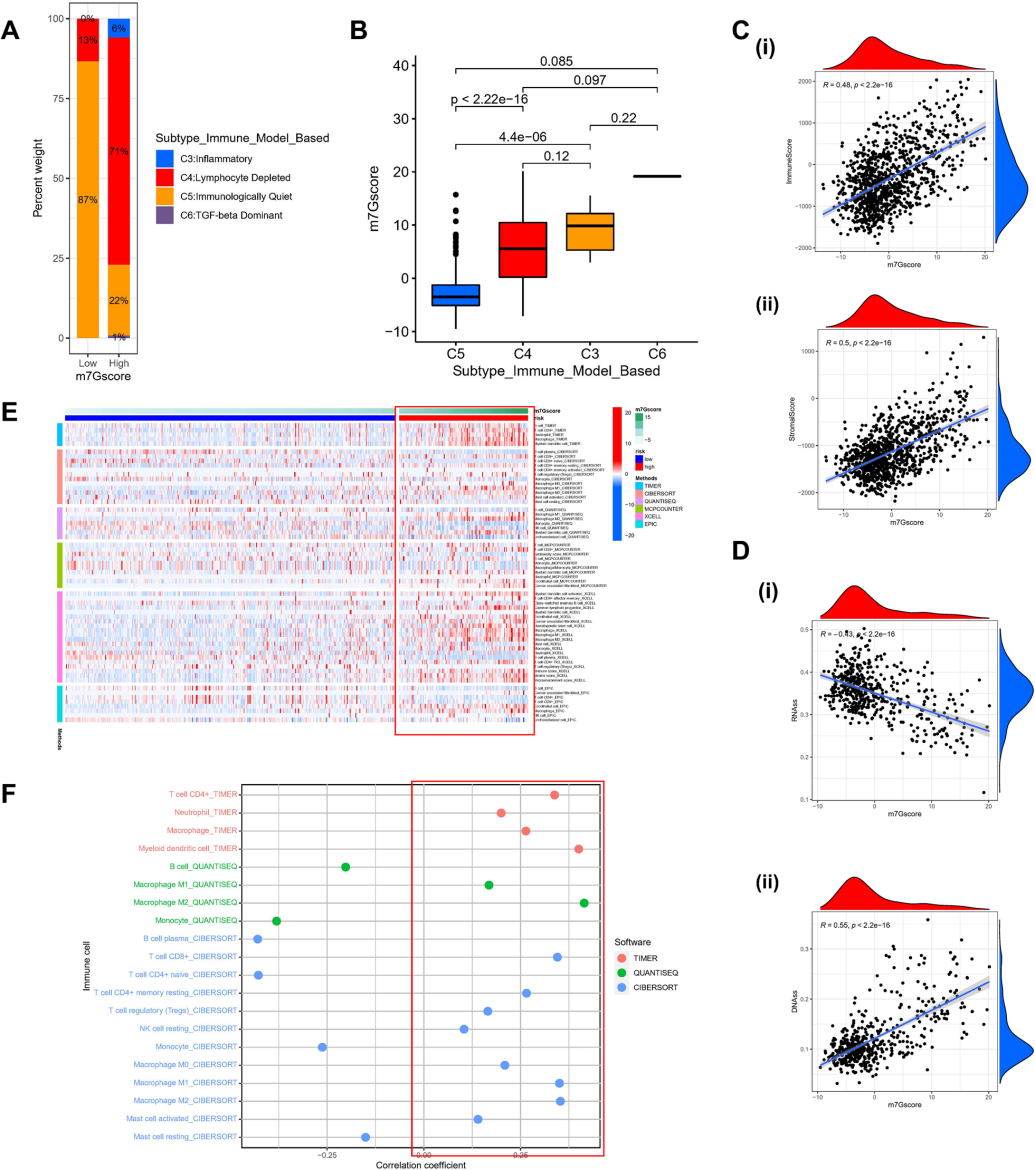
Identification of tumor immune microenvironment across high and low-risk m7G score groups. **A**. The proportion of LGG patients among C3 (inflammatory), C4 (lymphocyte depleted), C5 (immunologically quiet), and C6 (TGF-β Dominant) subtype immune model according to high- and low-risk m7G score. **B**. The comparison of the m7G score among C3、C4、C5、C6. **C**. (i) The correlation between m7Gscore and the Immune Score. (ii) The link between m7Gsocre and the stromal score. **D**. (i) The correlation between m7Gscore and the RNAs. (ii) The correlation between m7Gsocre and the DNAss (*p* < 0.001). **E**. Heatmap for immune responses by means of CIBERSORT, TIMER, QUANTISEQ, MCPCOUNTER, XCELL, and EPIC algorithms among high- m7Gscore group and low- m7Gscore group. **F**. The relationship between immune cells and m7Gscore. Each color represented a distinct algorithm

Based on the gene expression profiles of all solid tumors in TCGA, Thorsson et al. [32] established six immune expression signature subtypes: Wound Healing (Immune C1), IFN-gamma Dominant (Immune C2), Inflammatory (Immune C3), Lymphocyte Depleted(Immune C4), Immunologically Quiet (Immune C5), and TGF-beta Dominant (Immune C6). As per the aforementioned findings, we observed substantial variation of the immune subtypes among the various m7G score groups, with C4 dominating the high score group (Fig. 7A), and the scores also differed significantly among the immune subtypes (Fig. 7B). Stromal scores also increased with increasing m7G scores (Fig. 7C). In light of the critical role of stemness in tumor development and immunotherapy, we performed a correlation analysis of DNA and RNA stemness scores in LGG patients. Unsurprisingly, the m7G score was linearly related to the stemness score (Fig. 7D). To thoroughly explore the link between different m7G score subgroups and the immune microenvironment, we calculated the level of immune cell infiltration in each patient in the TCGA-LGG cohort following six algorithms: TIMER, CIBERSORT, QUANTISEQ, MCP-counter, XCELL, and EPIC, and found that in the high m7G score subgroup had more immune cell infiltration with TME in an activated state (Fig. 7E), and similarly, most immune cells were correlated positively with m7G score (Fig. 7F). Although most of the literature reports that LGG is insensitive to chemotherapy, we explored whether the m7G score-based grouping could indicate conventional cytotoxic drugs. Therefore, we calculated the IC50 values of various drugs using the prophetic package. It was found that most of the high m7G score groupings were more sensitive to chemotherapeutic drugs (Bleomycin, Cisplatin, Docetaxel, Etoposide, Gemcitabine), except Doxorubicin (Additional file 3: Fig S3).

### Screening the m7G scores hub genes and investigation of best-fitting compounds on hub genes

**Fig. 8:**
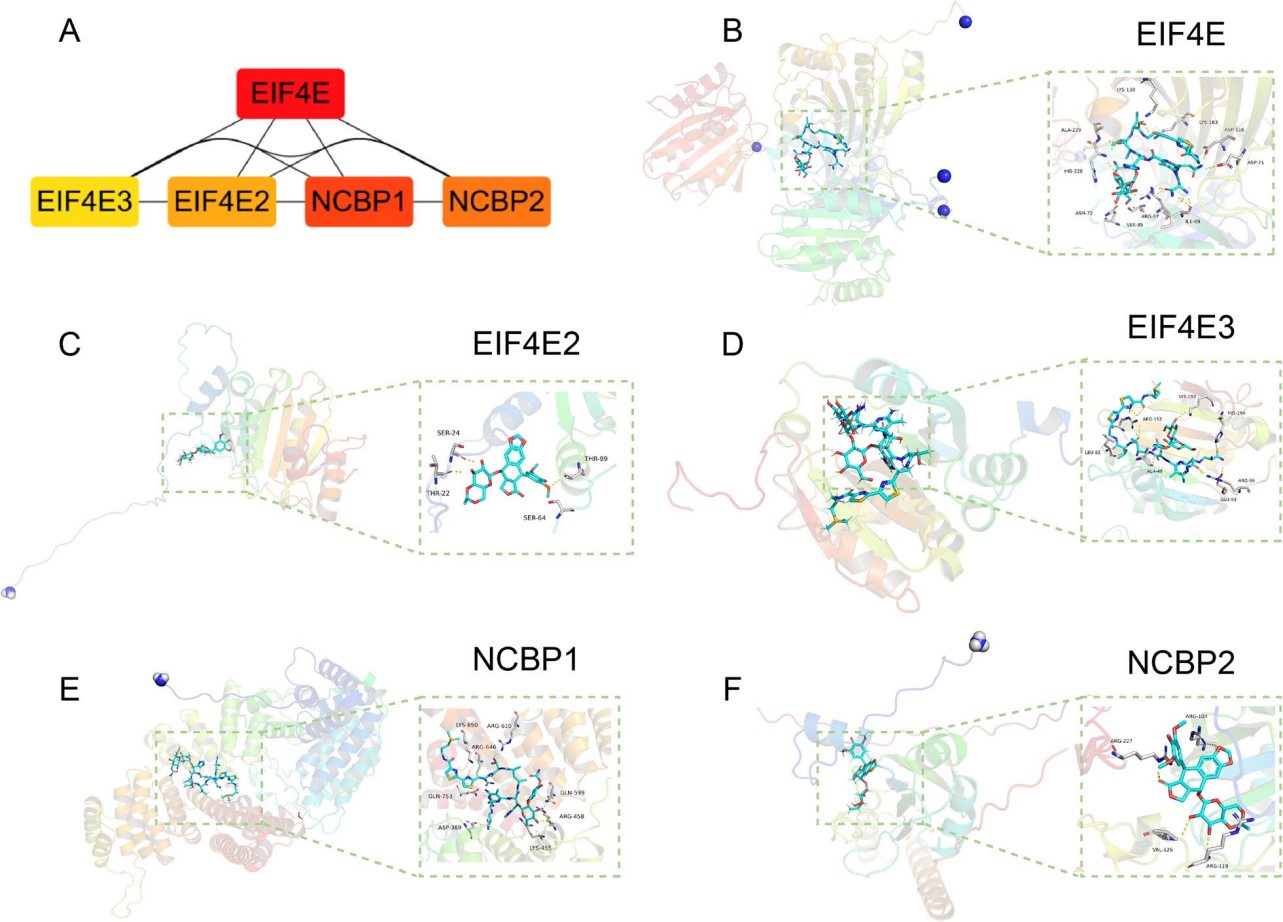
**A**. m7G score-hub gene network for the top 5 most highly regulated genes. **B**. Combination pattern diagram of Bleomycin and EIF4E. Yellow represents hydrogen bonding, and Amino acid residue includes ALA229, HIS228, ASN72, SER85, ARG87, ILE89, ASP71, ASP116, LYS183, and LYS138. **C**. Combination pattern diagram of Etoposide and EIF4E2. Yellow represents hydrogen bonding, Amino acid residue includes SER24, THR22, SER64, and THR99. **D**. Combination pattern diagram of Bleomycin and EIF4E3. Yellow represents hydrogen bonding, Amino acid residue includes ARG152, LEU83, ALA49, GLU93, ARG95, HIS194, and LYS192. **E**. Combination pattern diagram of Bleomycin and NCBP1. Yellow represents hydrogen bonding; Amino acid residue includes LYS650, ARG610, ARG646, GLN753, ASP369, LYS455, ARG458, and GLN599. **F**. Combination pattern diagram of Etoposide and NCBP2. Yellow represents hydrogen bonding, Amino acid residue includes ARG227, ARG104, VAL126, and ARG119. Notes: "Pocket" is a concave region made up of amino acid residues, the shape and chemistry of which allow other molecules to fit in and combine

We selected the top 5 genes with the highest connectivity (EIF4E, EIF4E3, EIF4E2, NCBP1, and NCBP2) to be considered as hub genes in m7G regulators (Fig. 8A). To investigate the most suitable compounds, we performed a virtual screening for molecular docking of these five genes using AutoDock Vina 1.1.2 (Fig. 8B–F). From a pharmaceutical perspective, different drugs have different affinities for critical targets. Bleomycin has a strong binding ability to each target, with each drug having a somewhat stronger affinity for EIF4E and NCBP1, with Bleomycin having a strong affinity for both, with total score values of 11.9795. Pymol visualization study revealed that Bleomycin bound to the cavity on the surface of EIF4E protein and formed hydrogen bonds with nine amino acid residues within the binding pocket, including LYS138, ALA229, HIS228, ASN72, SER85, ARG87, ILE89, ASP71, ASP116, and LYS183. Like the EIF4E protein, Bleomycin also binds to the cavity on the surface of the NCBP1 protein and hydrogen bonds with many amino acid residues within the cavity, including LYS650, ARG646, ARG610, GLN599, ARG458, LYS455, ASP369, and GLN753.

In terms of drug targeting, NCBP2 has a strong binding capacity with three components, Bleomycin (7.6277), Cisplatin (6.2128), and Gemcitabine (5.2287), with the most vital binding capacity with Bleomycin to NCBP2, and with NCBP2 within the binding pocket of ARG227, ARG104, VAL126, and ARG119 formed hydrogen bonds. NCBP1 had the strong binding ability with three components, Bleomycin (11.1593), Etoposide (5.9342), and Gemcitabine (5.1208), which had the strong binding ability with Bleomycin with NCBP2 with the strongest binding capacity. eIF4E3 had a strong binding capacity with Bleomycin (7.6016) and Etoposide (6.5918), with the strongest binding capacity with Bleomycin with NCBP2 and with EIF4E3 within the pocket of LEU83, ALA49, GLU93, ARG95, HIS194, LYS192, and ARG152 forming hydrogen bonding interaction. EIF4E has a strong binding ability with two components, Bleomycin (11.9795) and Etoposide (5.5992), with the strongest binding ability with Bleomycin to NCBP2. EIF4E2 has a strong binding ability with two components with strong binding ability, Bleomycin (5.1874) and Etoposide (5.316), with the strongest binding ability with Etoposide to NCBP2, forming hydrogen bonds with THR22, SER24, SER64, and THR99 within the binding pocket of EIF4E2.

### Expression validation of the m7G score hub genes by qRT-PCR

**Fig. 9:**
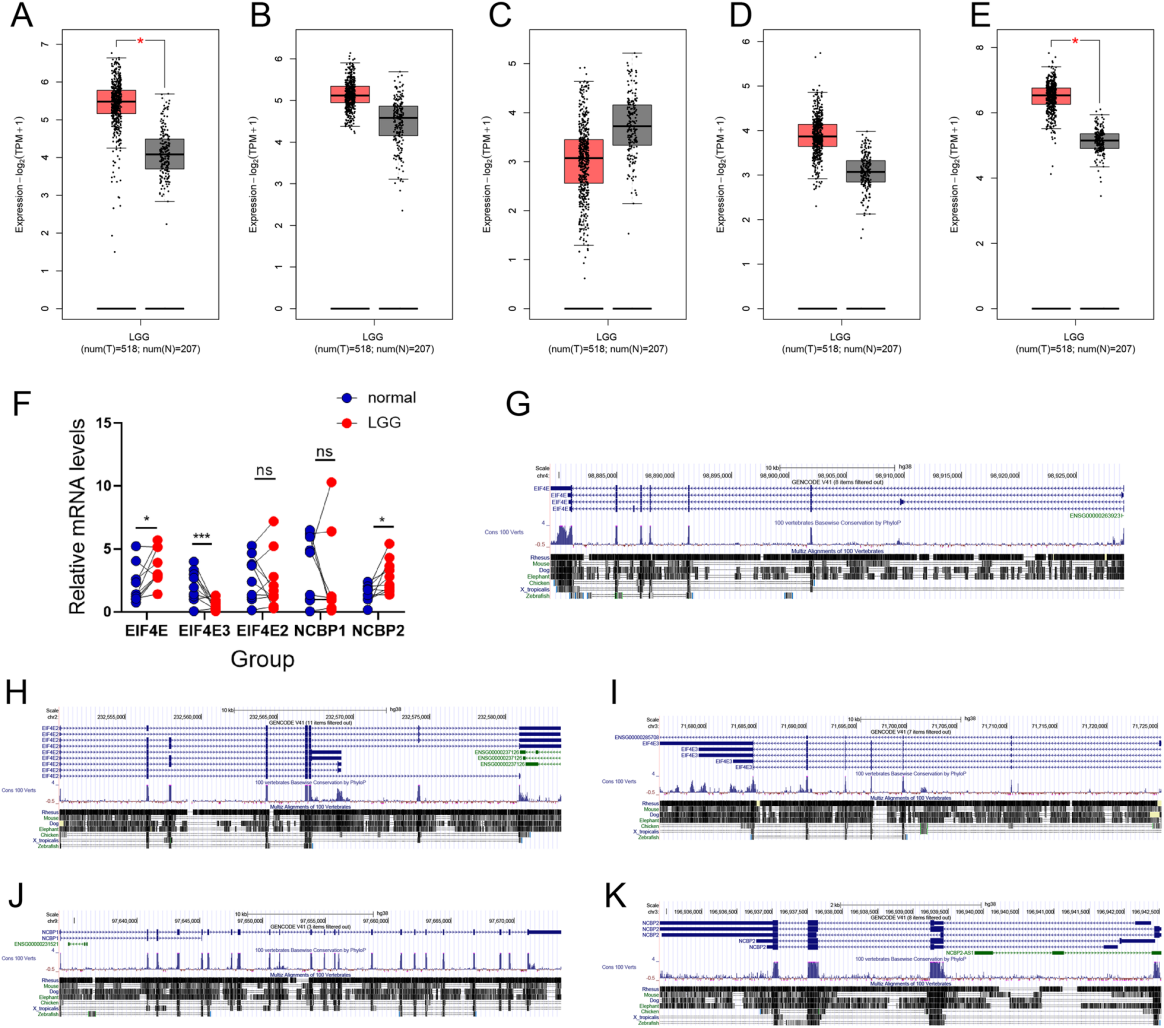
**A**–**E**. Comparison of the expression profiles of five hub genes (EIF4E, EIF4E3, EIF4E2, NCBP1, and NCBP2) between TCGA (518 LGG samples) and GTEx (207 healthy brain samples) cohorts by means of GEPIA. **F**. Bar plots representing the expression of five hub genes in LGG and healthy brain samples assessed by performing qRT-PCR (***p < 0.001, *p < 0.05). G-K. EIF4E, EIF4E2, EIF4E3, NCBP1, and NCBP2 gene conservation analysis among *Homo sapiens* was visualized using the UCSC genome browser

In total, five genes were examined using the GEPIA and GTEx databases to validate the expression status of the m7G Score hub genes(EIF4E, EIF4E3, EIF4E2, NCBP1, and NCBP2), and 207 healthy brain tissue samples and 518 LGG samples were found using the TCGA. According to the findings (Fig. 9A and E), the EIF4E and NCBP2 expression levels were considerably higher in the lower-grade glioma tissues in comparison to that in the healthy brain tissues (*p* < 0.05); while EIF4E2, EIF4E3, and NCBP1 expression levels in the lower-grade glioma tissues were not statistically different from normal brain tissues (Fig. 9B–D). Subsequently, for better characterization of the m7G Score hub gene’s expression levels in healthy and LGG tissues, 10 healthy brain tissue samples and 10 LGG samples were obtained. EIF4E, EIF4E3, and NCBP2 expression levels were substantially higher in LGG samples compared to healthy brain tissue samples (*p* < 0.05); whereas the EIF4E2 and NCBP1 expression levels in healthy brain tissues were not statistically significant with the expression in lower-grade glioma tissues (Fig. 9F), which is consistent with the results shown by GEPIA. Subsequently, the UCSC genome browser (https://www.genome.ucsc.edu/cgi-bin/hgGateway) was used to visualize gene conversation of EIF4E、 EIF4E3、 EIF4E2、 NCBP1、 and NCBP2 among homo sapiens according to the ChIPseq data of the ENCODE project (Fig. 9G–K).

### Western blot experiments

**Fig. 10:**
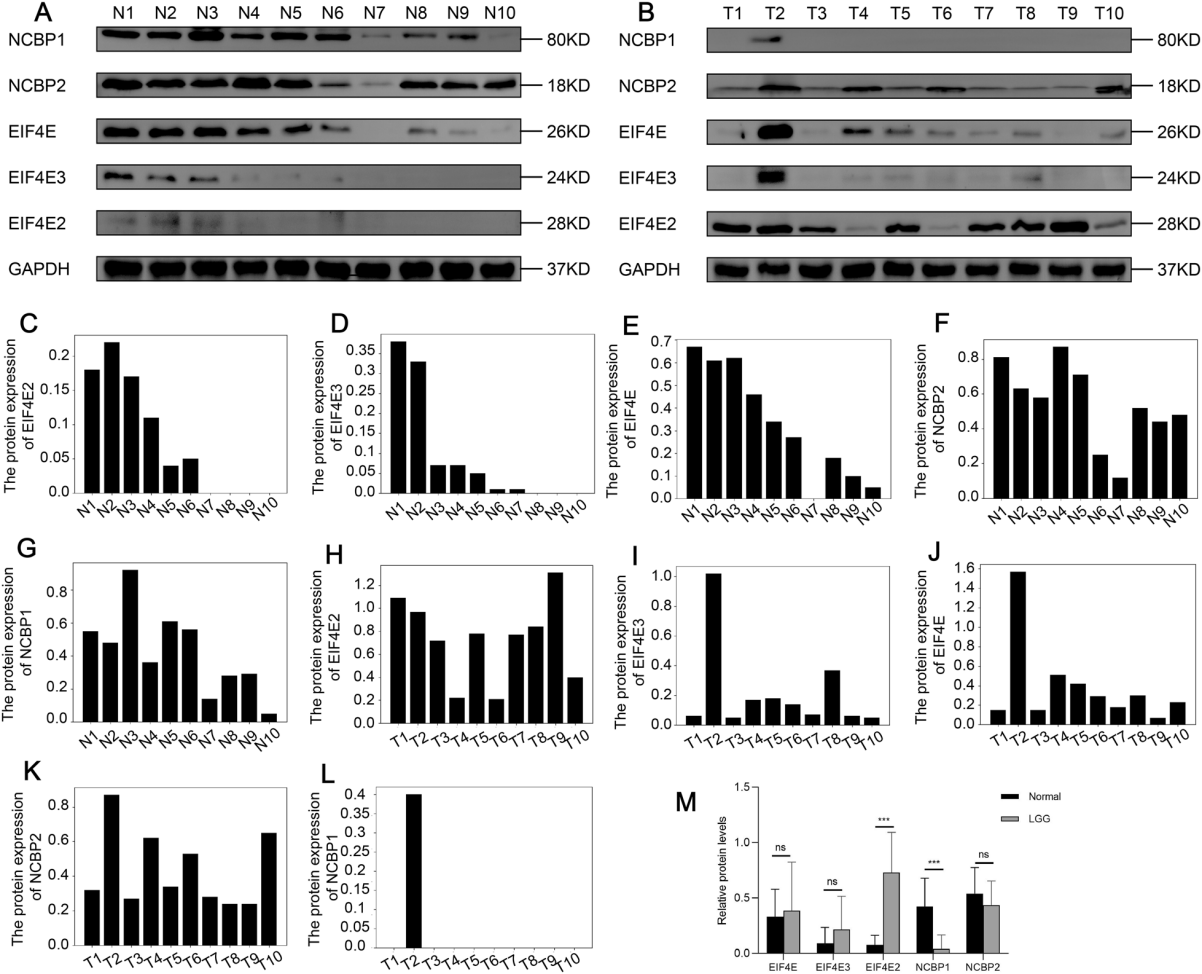
**A**–**B** Western blot experiment highlights the expression profile of NCBP1, NCBP2, EIF4E, EIF4E2, and EIF4E3 proteins in a total of ten tissue samples of LGG and ten healthy brain tissues. **C**–**M** Relative expression levels of NCBP1, NCBP2, EIF4E, EIF4E2, and EIF4E3 (five potentially prognostic m7G regulatory proteins) in ten LGG tissues and ten normal brain tissues. GAPDH was utilized as a loading control. The values were normalized by log2 fold change (ratio of tumor to healthy tissue expression) of the target proteins

The respective expression levels of NCBP1 and EIF4E2 proteins were lower and remarkably elevated in glioma tissues compared to the relatively healthy brain tissues, as per a Western blot analysis of 10 LGG tissues and 10 healthy brain tissues. On the other hand, there were no considerable differences in the protein levels of EIF4E, EIF4E3, and NCBP2 between healthy brain tissues and lower-grade glioma tissues (Fig. 10A–M). This was in line with the findings from the TCGA-LGG dataset-based bioinformatics analysis.

### Immunohistochemical experiments

**Fig. 11:**
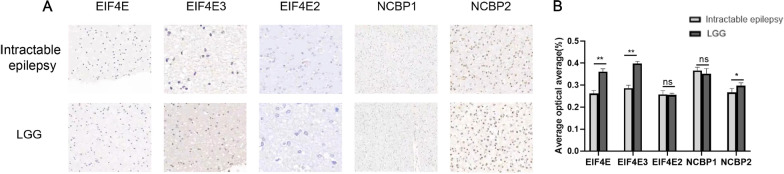
**A**, **B**. Immunohistochemistry (IHC) staining of intractable epilepsy tissues and LGG tissue using hematoxylin and eosin (HE) staining

In the subsequent steps, we collected 10 cases of intractable epilepsy tissues and 10 cases of lower-grade glioma (WHO II-III) tissues for immunohistochemical detection. Immunohistochemistry analysis showed that EIF4E, EIF4E3, and NCBP2 were increased in lower-grade glioma tissues, while lower-grade glioma did not cause a significant difference in EIF4E2 and NCBP1 proteins (Fig. 11A, B).

## Discussion

The m7G modifications are involved in gene regulation and tumor development [33]. Over- or under-expression of m7G regulators can alter m7G modifications in tumors and affect tumor progression [34]. Therefore, for treatment and patient prognosis of early tumor diagnosis, it is essential to study the molecular processes of m7G modification and to identify aberrant expression of m7G regulators in clinical, surgical specimens. The function of several m7G modulators in TME cell infiltration and the molecular mechanisms of the anti-tumor immune response are still unclear, despite the fact that the role of m7G in many cell types and microenvironments is beginning to emerge. For the purpose of characterizing TME cell infiltration in various m7G modification patterns, LGG immunotherapy was investigated. In a trial of PD-1/L1 inhibitors for the treatment of glioma, Cloughesy Timothy F et al. [35] found that the neoadjuvant PD-1 blockade administration promotes the local and systemic antitumor immune response and could be a more effective strategy for treating this consistently fatal brain tumor. In addition, PD-1 neoadjuvant therapy similarly modulated the tumor immune microenvironment in a single-arm phase II clinical trial(NCT02550249) aiming to examine the feasibility, safety, and immunobiological effects of PD-1 blockade in individuals undergoing surgery for glioblastoma [36], resulting in elevated cytokine expression, increased immune cell tumor infiltration, and clonal expansion of tumor-infiltrating T cells.

Therefore, an in-depth study of the role of PD-1/L1 in tumors is needed to improve the benefits of therapy. For example, in our anti-PD-1 cohort study, individuals with a high m7G Score showed significant clinical benefit and significantly prolonged survival. However, no considerable differences in immunotherapy outcomes among individuals in different m7G Score subgroups were observed. Interestingly, in the anti-PD-L1 cohort, individuals with low m7G Scores had better prognostic outcomes and were more inclined to CR/PR. As a result, evaluating m7G modification patterns is a promising and reliable biomarker for evaluating patient prognosis and relevant clinical outcomes following immunotherapy. PD-1 or PD-L1 response may be represented by the differential status of m7G alteration, which may also aid in predicting the efficacy of anti-PD-1/L1 Immunotherapy and PD-1/L1 immunotherapy.

Additionally, using patterns of m7G methylation modification, we identified DEGs. Additional investigation revealed that these DEGs contained signature genes related to the m7G pattern and tumor prognosis. These m7G pattern-associated signature genes were genotyped based on cluster analysis. ssGSEA results indicated that they were closely associated with significantly enhanced hallmark pathway activity. After that, adjusting mRNA levels in accordance with the m7G methylation modified genes, we classified them into groups with high and low expression based on median mRNA expression. Furthermore, the m7G riskScore was developed to assess the m7G modification pattern of different LGG patients and to counteract the impact of individual heterogeneity. In individuals with LGG, this offered an accurate guide foriImmunotherapy. Numerous studies have shown that high TMB is closely related to clinical benefits and prolonged survival in patients [37–39]. To aid clinical decision-making, TMB can be employed as a predictive biomarker for LGG patients. The results of our study showed that the m7G score was substantially and positively correlated with TMB, i.e., lower TMB scores in the Low-m7G score group and Low-TMB grouping represented better prognostic outcomes, and when the Low-m7G score combined with Low-TMB indicated better prognostic outcomes, which further validated the predictive advantage of m7G score in immunotherapy for LGG patients. In addition, we identified six drugs with significant sensitivity in the prognostic model (Bleomycin, Cisplatin, Docetaxel, Etoposide, and Gemcitabine) that may improve the clinical outcome of LGG.

Numerous studies have found that m7G regulator hub genes (EIF4E, EIF4E3, EIF4E2, NCBP1, and NCBP2) are crucial in tumor progression and metastasis. EIF4E is a key translation messenger ribonucleic acid (mRNA) to protein initiator in eukaryotic cells, previously Dr. Ruggero et al. [40] found that EIF4E ± mice containing only one copy incubated by gene editing in mice, although expressing only 50% of the amount of EIF4E, do not affect the overall mRNA translation of the mice but rather target the expression of only a specific class of genes, especially those in some oncogenic pathways. The EIF4E-Sox2 axis has also been demonstrated to represent a novel mechanism for unmodulated self-renewal of glioma-initiating cells, offering a potential therapeutic target for glioma [41]. Using a particular structural pose, Frosi Yuri et al. showed how molecules interact with eIF4E at the eIF4G binding site [42]. In addition, it has been shown that the upregulation of eukaryotic translation initiation factor 4E enhances cell proliferation both in vitro and in vivo and is linked to an unsatisfactory prognosis in cases of gallbladder cancer [43]. In addition, High EIF4E2 expression has been shown by Yang et al. to be an independent prognostic risk factor for UM patients. During the course of UM, EIF4E2 may be crucial in hypoxia-related signaling pathways [44]. A regulatory mechanism for this potential tumor suppressor in the inhibition of HIF-2- and eIF4E2-mediated translation activation of oncogenic mRNAs was discovered by other researchers, who found that DDX28 (DEAD Box Protein Family Member) is a Negative Regulator of Hypoxia-Inducible Factor 2α- and Eukaryotic Initiation Factor 4E2-Directed Hypoxic Translation [45]. Melanson Gaelan et al. found the EIF4E2-Directed Hypoxic Cap-Dependent Translation Machinery Reveals Novel Therapeutic Potential for Cancer Treatment [46]. eIF4E3 is a core component of translation initiation and regulation in eukaryotic cells and can selectively control the translation and expression of oncogenes. eIF4E3 is a newly identified potential oncogene. According to prior studies, overexpression of eIF4E3 can compete for target mRNAs that bind eIF4E and prevent the translation of factors such as VEGF, suggesting that eIF4E3 is a potential oncogene against eIF4E [47]. Landon, AL et al. [48] further found that in addition to inhibiting the function of eIF4E1, eIF4E3 can initiate the translation of n-Myc, HMGA, CDX2, Twist, etc. which are usually not the target genes of eIF4E1, suggesting that eIF4E3 can initiate the translation of target genes different from eIF4E1.

Nuclear cap-binding proteins NCPB1 and NCPB2 (known in the literature as cap-binding protein 80 [CBP80] and CBP20, respectively, based on their molecular weights) form heterodimers to produce nuclear CBC, which is largely conserved from plants to humans [49]. Strong evidence revealed that NCPB1 and NCPB2 engage in transcription, splicing, transcriptional output and translation, and the processing of histone RNA15 and mammalian spliceosome [50]. In addition, one researcher extensively investigated the function of NCBP1 in lung cancer cell proliferation and migration using two lung cancer cell lines with NCBP1 knockdown and overexpression. H1299 cells' proliferation and migration were inhibited when NCBP1 was downregulated, whereas the effect was contrary when NCBP1 was subjected to overexpression. These findings imply that lung cancer cell proliferation and migration may be at least facilitated by NCBP1 [51]. In addition, hypoxic tumor-associated fibroblasts increase NCBP2-AS2/HIAR through an enhanced VEGF signaling pathway and promote endothelial cell sprouting [52].

RT-qPCR was conducted to explore the expression of hub genes, revealing that, with the exception of EIF4E3, our samples shared a similar expression pattern with the samples from the public database. Western blot analysis showed that the protein expression levels of EIF4E2 and NCBP1 were significantly elevated in glioma tissues compared to matched healthy brain tissues. In contrast, immunohistochemical analysis of 10 refractory epilepsy tissues and 10 lower-grade gliomas (WHO grade II-III) tissues showed that lower-grade gliomas did not significantly increase in EIF4E2 and NCBP1 protein expression, while EIF4E, EIF4E3, and NCBP2 were increased. The value of the m7G score in clinical practice for individuals with LGG is demonstrated by a systematic study of the score. The m7G score can be utilized to analyze the TME cell infiltration status that corresponds to the m7G methylation pattern in LGG patients, which can improve our understanding of the immunophenotype of LGG and enhance the translational impact of therapy. Moreover, the m7G score could serve as an independent predictive biomarker for LGG, providing additional criteria for clinical treatment and evaluating the clinical outcomes of immunotherapy. Importantly, this study confirms the role of m7G regulators or m7G phenotype-associated genes in LGG, offering new avenues for epigenetic and oncological studies and potential regulatory mechanisms.

While we explored the transcriptional link between m7G methylation modifications and LGG in our study, it is important to note that not all m7G methylation alterations are fully reflected in transcriptional changes. Other factors, such as protein modification states, also play a crucial role. As such, additional studies are necessary, along with histology data, to fully comprehend the relationship between m7G methylation modifications and LGG.

## Conclusion

In summary, the present investigation thoroughly assessed the modification patterns of 23 m7G regulators in LGG. It was thus shown that various modification patterns might play a significant role in the variability and heterogeneity of TMB inhibition. Based on an analysis of m7G modification patterns, this will clarify the TMB infiltration features of LGG patients and their role in PD-1/L1. In addition to providing opportunities for prognosis prediction and investigation of new immunotherapies in clinical LGG patients, this encourages basic research in related fields.

### Supplementary Information

**Additional file 1: Figure S1.** A. Kaplan–Meier survival analysis to predict the OS of individuals with LGG based on the 20 m7G-related genes.

**Additional file 2: Figure S2.** A. (i) The proportion of LGG patients Age < 40 and Age > = 40 in high- and low- m7G score. (ii-iii) Kaplan–Meier curve analysis of OS in the high- and low- m7G score for patients in the two age groups. (< 40 and > = 40 years). B. (i) The proportion of LGG patients Female and Male in high- and low- m7G score. (ii–iii) Kaplan–Meier curve analysis of OS in the high- and low- m7G score for patients in the two gender groups. (female and male). C. (i–iv) Kaplan–Meier curve analysis for OS in high- and low-m7G score for patients in the EGFR Mutation、EGFR Wild、TP53 Mutation, and TP53 Wild groups.

**Additional file 3: Figure S3.** A–F. The half-maximal inhibitory concentration (IC50) of 6 widely used chemotherapeutic drugs (Bleomycin, Cisplatin, Docetaxel, Etoposide, Gemcitabine, and Doxorubicin).

**Additional file 4: Figure S4.** A–B. Cox regression assessment on univariate and multivariate data in the TCGA dataset and CGGA dataset, along with the model, indicating outstanding prognostic ability independent of clinicopathological variables. C. m7G score distribution, patient survival status and time, and heatmap of the m7G score in low and high-score. D. The relationship between m7Gscore and the OS.

## Data Availability

The original contributions presented in the study are included in the article/Supplementary Material, further inquiries can be directed to the corresponding author.

## Abbreviations

m7G: N7-methylguanosine
LGG: Lower-grade glioma
CGGA: The Chinese Glioma Genome Atlas
TCGA: The Cancer Genome Atlas
GSEA: Gene set enrichment analysis
ssGSEA: Single sample GSEA
PCA: Principal component analysis
CR: Complete response
PR: Partial response
TMB: Tumor mutational burden
IDH: Isocitrate dehydrogenase
TME: Tumor microenvironment
PD-1: Programmed cell death protein 1
PD-L1: Programmed cell death protein ligand 1
MDSCs: Myeloid-derived suppressor cells
CBP80: Cap-binding protein 80
