# Correction: N7‑methylguanosin regulators‑mediated methylation modification patterns and characterization of the immune microenvironment in lower‑grade glioma

**DOI:** 10.1186/s40001-024-01656-3

**Published:** 2024-01-19

**Authors:** Aierpati Maimaiti, Zhaohai Feng, Yanwen Liu, Mirzat Turhon, Zhihao Xie, Yilimire Baihetiyaer, Xixian Wang, Maimaitijiang Kasimu, Lei Jiang, Yongxin Wang, Zengliang Wang, Yinan Pei

**Affiliations:** 1 Department of Neurosurgery, Neurosurgery Centre, The First Affiliated Hospital of Xinjiang Medical University, No. 137, South Liyushan Road, Xinshi District, Urumqi, 830054 Xinjiang China; 2 Department of Medical Laboratory, Xinjiang Production and Construction Corps Hospital, Urumqi, 830002 Xinjiang China; 3 Department of Neurointerventional Surgery, Beijing Neurosurgical Institute, Capital Medical University, Beijing, 100070 China; 4 Department of Neurointerventional Surgery, Beijing Tiantan Hospital, Capital Medical University, Beijing, 100070 China; 5 The Second Hospital of Jilin University, Changchun, 130041 Jilin China; 6 Department of Neurology, The First Affiliated Hospital of Xinjiang Medical University, Urumqi, 830054 Xinjiang China; 7 People’s Hospital of Mongolian Autonomous Prefecture of Bayingolin, Korla, 841000 Xinjiang China

**Correction: European Journal of Medical Research (2023) 28:144** 10.1186/s40001-023-01108-4

After online publication [1], the authors identified that Fig. 10 contains a mistake in the graphic combination, which has resulted in two Western blot prints being repeated in the same figure. For the sake of aesthetics, the authors had opened the AI with the same WB strip name for the two groups. When the PS file was re-imported, the PS file with the same name was placed into the AI image, resulting in the coincidence of the two groups. The corrected Fig. 10 is given in this Correction article.

**Fig. 10:**
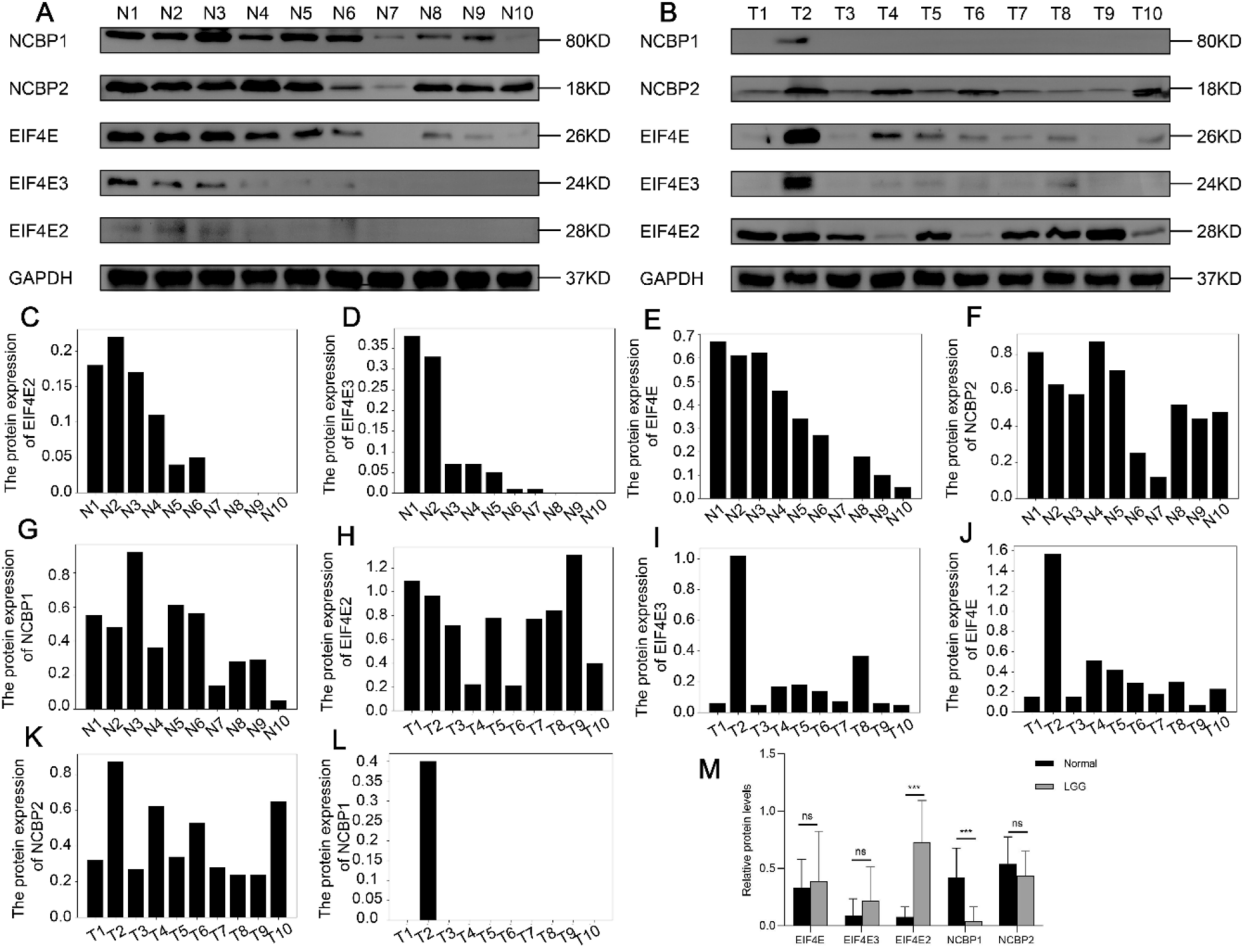
**A**, **B** Western blot experiment highlights the expression profile of NCBP1, NCBP2, EIF4E, EIF4E2, and EIF4E3 proteins in a total of ten tissue samples of LGG and ten healthy brain tissues. **C**–**M** Relative expression levels of NCBP1, NCBP2, EIF4E, EIF4E2, and EIF4E3 (five potentially prognostic m7G regulatory proteins) in ten LGG tissues and ten normal brain tissues. GAPDH was utilized as a loading control. The values were normalized by log2 fold change (ratio of tumor to healthy tissue expression) of the target proteins

The legend of Fig. 8 was incompletely published in the original version. The corrected legend of Fig. 8 to avoid confusion about “pockets” was given below.

**Figure 8 legend:**

**A** m7G score-hub gene network for the top 5 most highly regulated genes. **B** Combination pattern diagram of Bleomycin and EIF4E. Yellow represents hydrogen bonding, and Amino acid residue includes ALA229, HIS228, ASN72, SER85, ARG87, ILE89, ASP71, ASP116, LYS183, and LYS138. **C** Combination pattern diagram of Etoposide and EIF4E2. Yellow represents hydrogen bonding, Amino acid residue includes SER24, THR22, SER64, and THR99. **D** Combination pattern diagram of Bleomycin and EIF4E3. Yellow represents hydrogen bonding, Amino acid residue includes ARG152, LEU83, ALA49, GLU93, ARG95, HIS194, and LYS192. **E** Combination pattern diagram of Bleomycin and NCBP1. Yellow represents hydrogen bonding; Amino acid residue includes LYS650, ARG610, ARG646, GLN753, ASP369, LYS455, ARG458, and GLN599. **F** Combination pattern diagram of Etoposide and NCBP2. Yellow represents hydrogen bonding, Amino acid residue includes ARG227, ARG104, VAL126, and ARG119. Notes: “Pocket” is a concave region made up of amino acid residues, the shape and chemistry of which allow other molecules to fit in and combine.

The original article has been corrected.
