# Supplementary figures and images for: N7-methylguanosin regulators-mediated methylation modification patterns and characterization of the immune microenvironment in lower-grade glioma
Source: Eur J Med Res. 2023 Mar 30;28:144. doi: 10.1186/s40001-023-01108-4 (PMC10061823; doi:10.1186/s40001-023-01108-4)

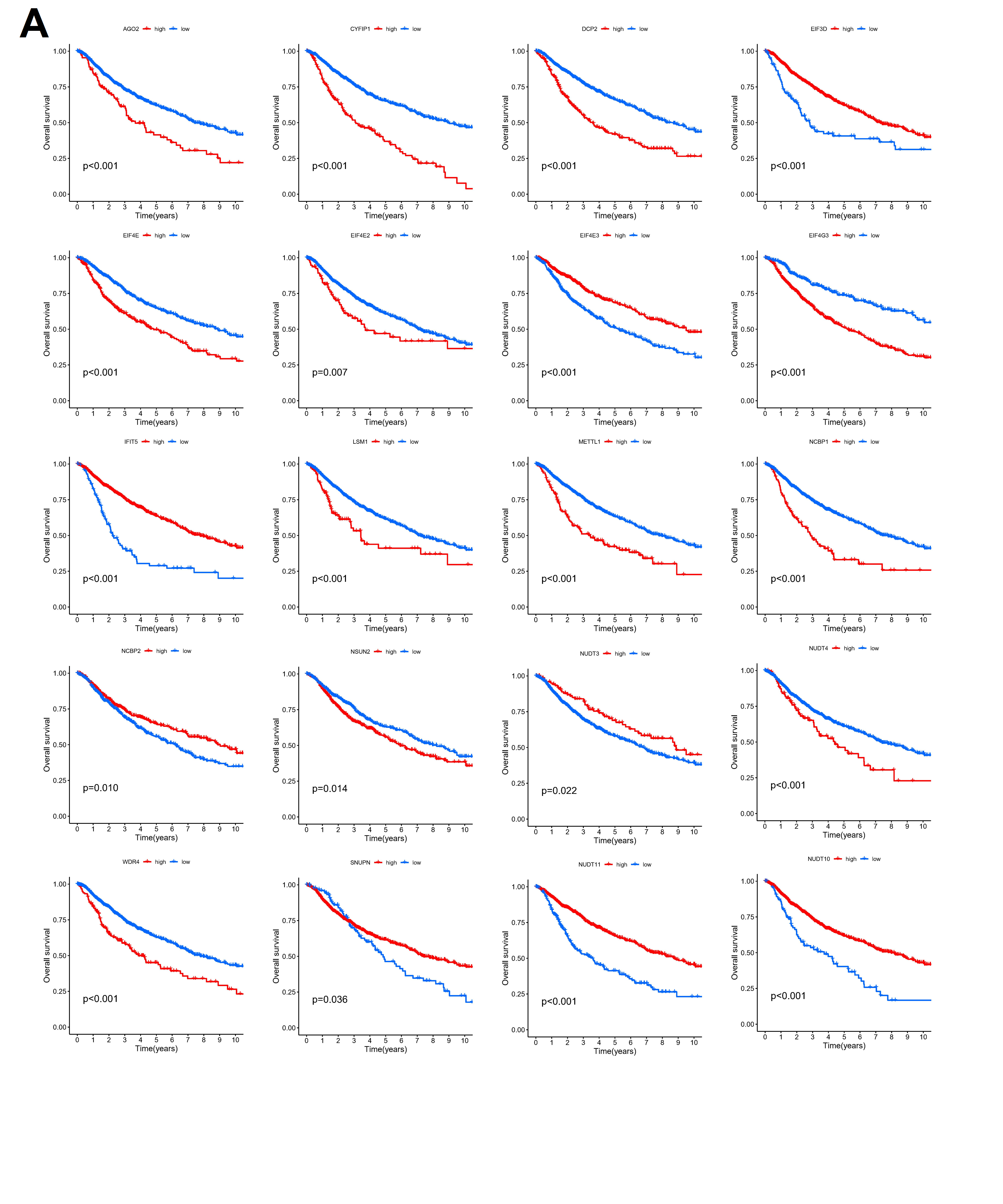

Supplement: Supplementary file 1 — Additional file 1: Figure S1. A. Kaplan–Meier survival analysis to predict the OS of individuals with LGG based on the 20 m7G-related genes. [file 40001_2023_1108_MOESM1_ESM.tiff]

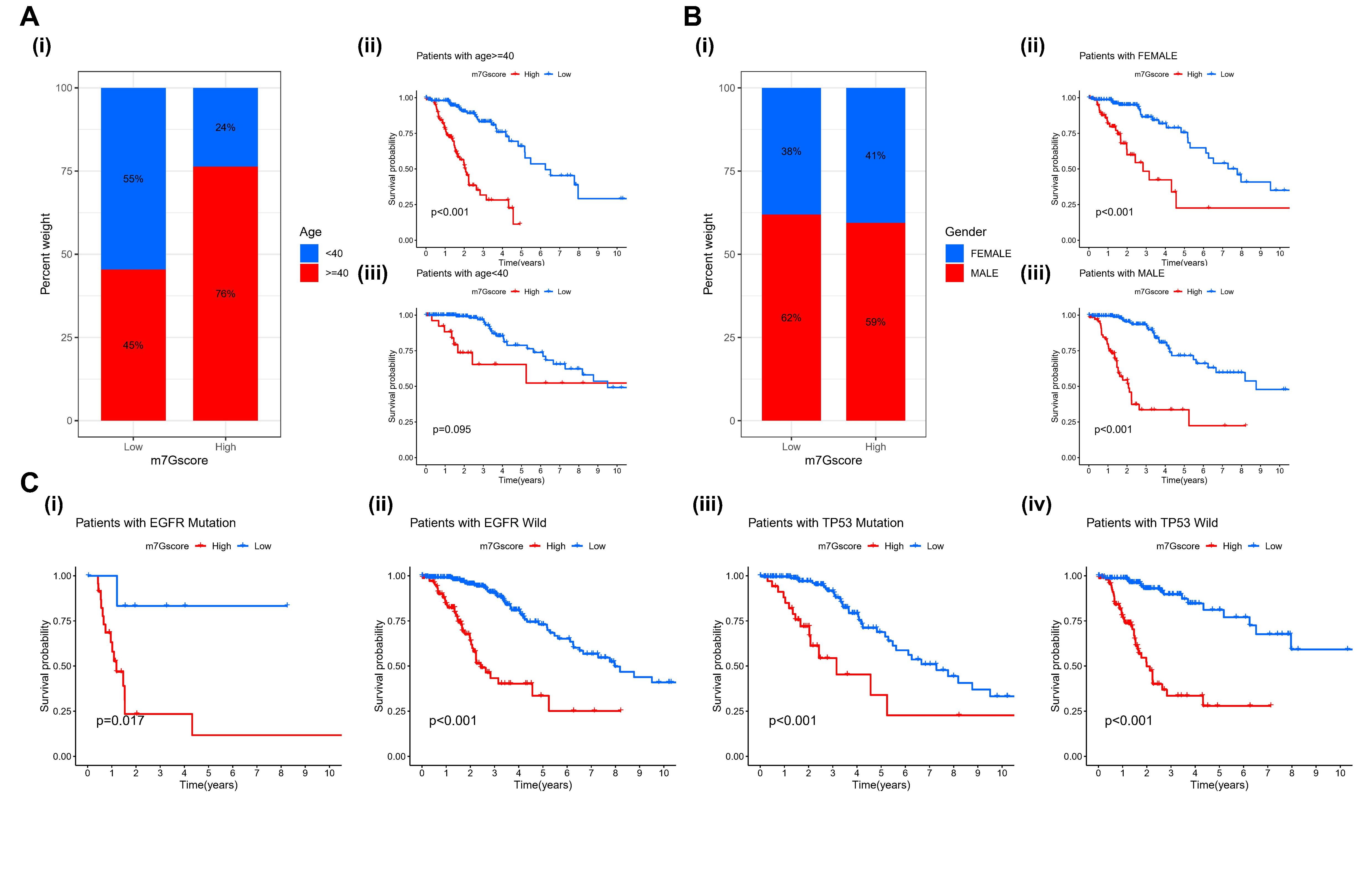

Supplement: Supplementary file 2 — Additional file 2: Figure S2. A. (i) The proportion of LGG patients Age < 40 and Age > = 40 in high- and low- m7G score. (ii-iii) Kaplan–Meier curve analysis of OS in the high- and low- m7G score for patients in the two age groups. (< 40 and > = 40 years). B. (i) The proportion of LGG patients Female and Male in high- and low- m7G score. (ii–iii) Kaplan–Meier curve analysis of OS in the high- and low- m7G score for patients in the two gender groups. (female and male). C. (i–iv) Kaplan–Meier curve analysis for OS in high- and low-m7G score for patients in the EGFR Mutation、EGFR Wild、TP53 Mutation, and TP53 Wild groups. [file 40001_2023_1108_MOESM2_ESM.tiff]

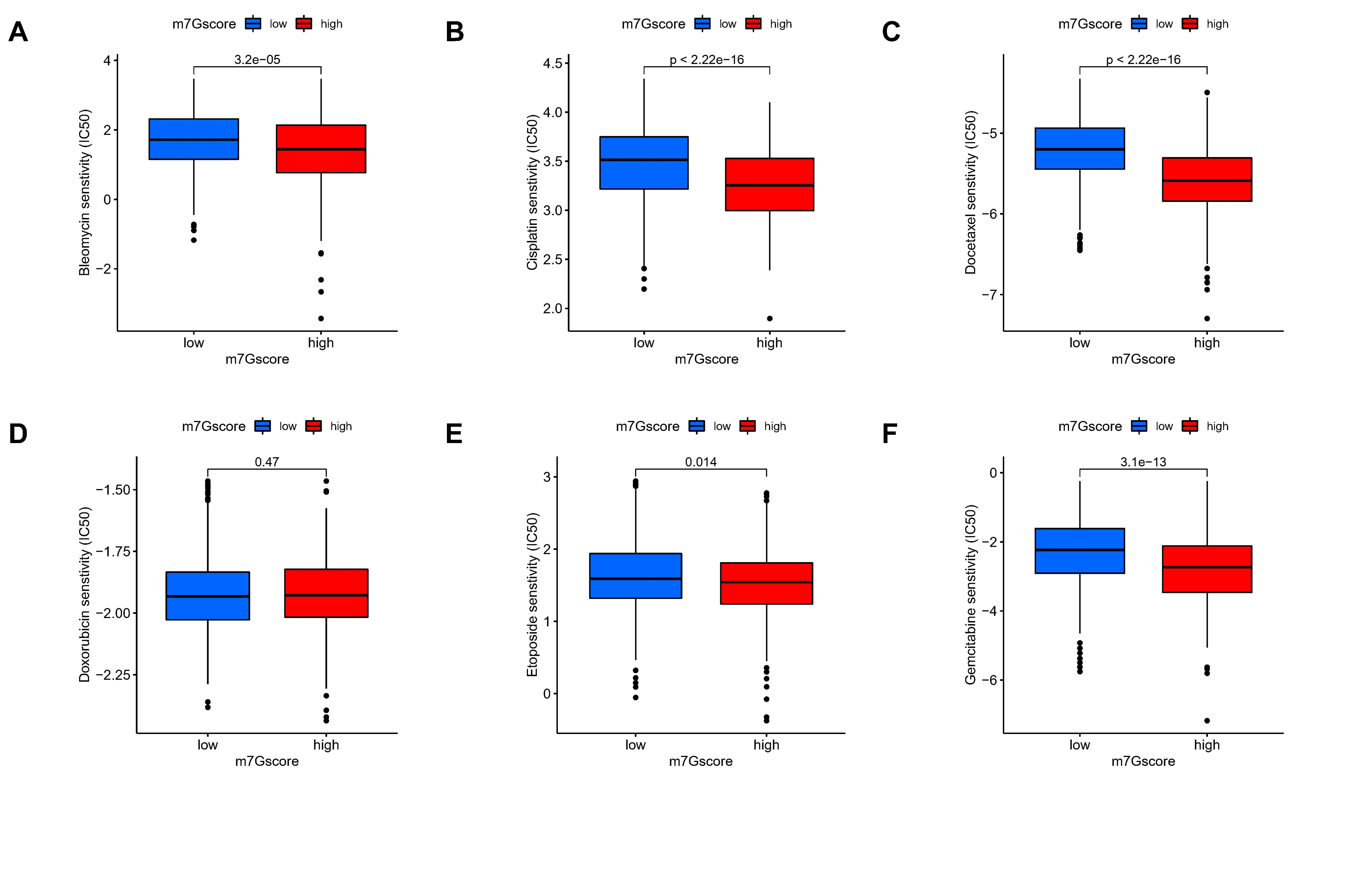

Supplement: Supplementary file 3 — Additional file 3: Figure S3. A–F. The half-maximal inhibitory concentration (IC50) of 6 widely used chemotherapeutic drugs (Bleomycin, Cisplatin, Docetaxel, Etoposide, Gemcitabine, and Doxorubicin). [file 40001_2023_1108_MOESM3_ESM.tiff]

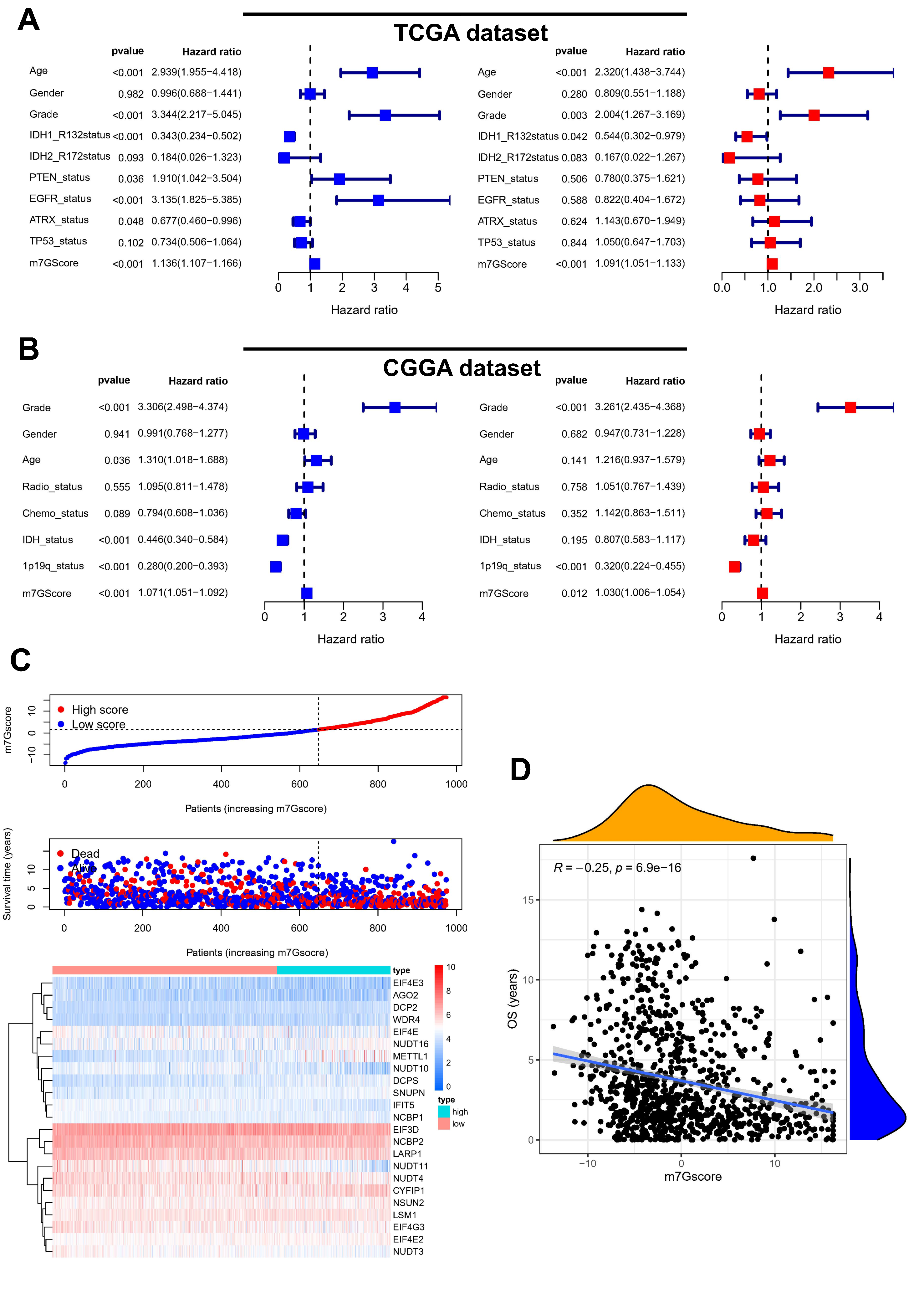

Supplement: Supplementary file 4 — Additional file 4: Figure S4. A–B. Cox regression assessment on univariate and multivariate data in the TCGA dataset and CGGA dataset, along with the model, indicating outstanding prognostic ability independent of clinicopathological variables. C. m7G score distribution, patient survival status and time, and heatmap of the m7G score in low and high-score. D. The relationship between m7Gscore and the OS. [file 40001_2023_1108_MOESM4_ESM.tiff]
